# The impact of social structure on breeding strategies in an island bird

**DOI:** 10.1038/s41598-020-70595-w

**Published:** 2020-08-17

**Authors:** Grant C. McDonald, Noémie Engel, Sara S. Ratão, Tamás Székely, András Kosztolányi

**Affiliations:** 1 Department of Ecology, University of Veterinary Medicine Budapest, Budapest, Hungary; 2 Department of Zoology, Edward Grey Institute, University of Oxford, Oxford, UK; 3 Department of Biology and Biochemistry, Milner Centre for Evolution, University of Bath, Bath, UK; 4 FMB, Fundação Maio Biodiversidade, Cidade do Porto Inglês, Maio, 6110 Cabo Verde; 5 Department of Evolutionary Zoology and Human Biology, University of Debrecen, Debrecen, Hungary

**Keywords:** Behavioural ecology, Evolutionary ecology, Animal behaviour

## Abstract

The social environment is a key factor determining fitness by influencing multiple stages of reproduction, including pair formation, mating behavior and parenting. However, the influence of social structure across different aspects of breeding is rarely examined simultaneously in wild populations. We therefore lack a consolidation of the mechanisms by which sociality impacts reproduction. Here we investigate the implications of the social environment before and during breeding on multiple stages of reproduction in an island population of the ground nesting shorebird, the Kentish plover (*Charadrius alexandrinus*). We utilise information on mating decisions, nest locations and nesting success across multiple years in combination with social network analysis. Sociality before breeding was connected with patterns of pair formation. In addition, site fidelity and personal breeding experience was associated with the spatial organisation of breeding pairs. Our results provide evidence that, while differential social interactions at localised scales influence patterns of reproductive pairing, site fidelity and personal breeding experience influence the structure of populations at the landscape scale. Our results underline the tight link between the social structure of populations and patterns of mating, while revealing that the relative influence of sociality, breeding experience and local ecology are dynamic across different facets of reproduction.

## Introduction

The breeding success of individuals is shaped by a complex interplay of environmental factors including predation, parasite abundance, precipitation and food availability^1–6^. The influence of these factors can vary across multiple stages of reproduction from courting and mating to parental care^7–11^. In addition to the aforementioned factors, the social environment is a key factor affecting reproduction, and has been the focus of recent intense research associated with the growth of social network analyses^12–16^. An individual’s social environment describes the phenotypic composition of interacting con- and hetero-specifics and their rates of interactions^17–20^, and influences key population processes including the flow of information and diseases, as well as rates of cooperation and conflict^21–25^. Understanding the repercussions of the social environment on individual breeding success is therefore of key importance for predicting the evolutionary and demographic trajectories of populations^23,24,26,27^.

The social environment may influence breeding success through a variety of mechanisms. For example, the social environment before mating can shape the availability of suitable mates, rates of sexual harassment, and the intensity of intrasexual competition^13,15,28–32^. Patterns of sociality before breeding may also reflect precopulatory strategies to attain mates and impact mate choice decisions^33–35^. For example, socialisation before breeding may allow females to assess the quality of potential mates^36^. Moreover, socialisation between reproductive pairs before reproduction may impact subsequent breeding success, if increased familiarity between males and females shapes pair-bond strength and improves the coordination of breeding behaviour between partners^28,37^. Alternatively, the social environment could impact breeding success after the formation of reproductive pairs, by influencing the timing and choice of breeding territories. For example, if familiarity between territorial neighbours reduces conflict, individuals who socialise more prior to breeding may select contiguous breeding territories^38–40^. Similarly, more close social associations with conspecifics may facilitate synchronized behavior across breeding pairs, benefiting individuals through increasing the efficiency of social foraging, access to social information, vigilance, nest defense and reduced predation via “dilution effects”^41^. Further still, breeding structure may impact future breeding decisions if public information obtained from the social environment during breeding influences decisions over breeding dispersal. For example, in several species of colonial birds, individuals assess the quality of breeding sites through the breeding success of local breeding neighbours^42–44^. Understanding the mechanisms through which social structure impacts reproduction thus requires detailed information across multiple stages of breeding and remains an outstanding goal.

Here we utilise information on pre-breeding sociality in the ground-nesting shorebird the Kentish plover (*Charadrius alexandrinus*) in combination with information on mating decisions, nest locations, breeding dispersal and nesting success, to assess the impact of social structure on breeding ecology across multiple stages of reproduction. The breeding system of Kentish plover has been intensively studied, plovers typically form socially monogamous pairs and parents cooperate to incubate nests^45–50^. Once broods hatch, offspring may be cared for by both parents, or following desertion, uniparental care may follow, after which deserting parents may subsequently remate^50^. Characterising social structure and social behaviour is often challenging in migratory and colonial breeding populations, where little is known of social interactions between individuals in locations away from breeding sites^41^. We overcome this challenge by studying a resident island population, facilitating the collection of information on social associations year-round.

We firstly examine the role of social structure before breeding in shaping patterns of reproductive pairing. We predict that if pre-breeding social structure affects mate choice then pre-breeding sociality should correlate with patterns of reproductive pairing. In addition, if pair-bond strength and parental coordination is influenced by greater social familiarity, we predict that pairs who associate more strongly before breeding should have higher subsequent breeding success. Secondly, we link pre-breeding sociality to the organisation of breeding colonies. We predict that if the spatial organisation of breeding territories has implications for nest success through conflict (or cooperation) between breeding neighbours, then individuals that are more familiar during the non-breeding season should nest closer together and the density of breeding neighbours will impact on nesting success. Moreover, if increased sociality is related to increased social information, social connectivity should be related to the breeding synchrony of individuals. Finally, we explore how social structure during breeding may influence future decisions over breeding dispersal. We predict that if individuals use social information from local breeding neighbours, pairs with less successful local breeding neighbours should be more likely to nest in a different location for their subsequent nesting attempt.

## Methods

### Study site and population

**Figure 1:**
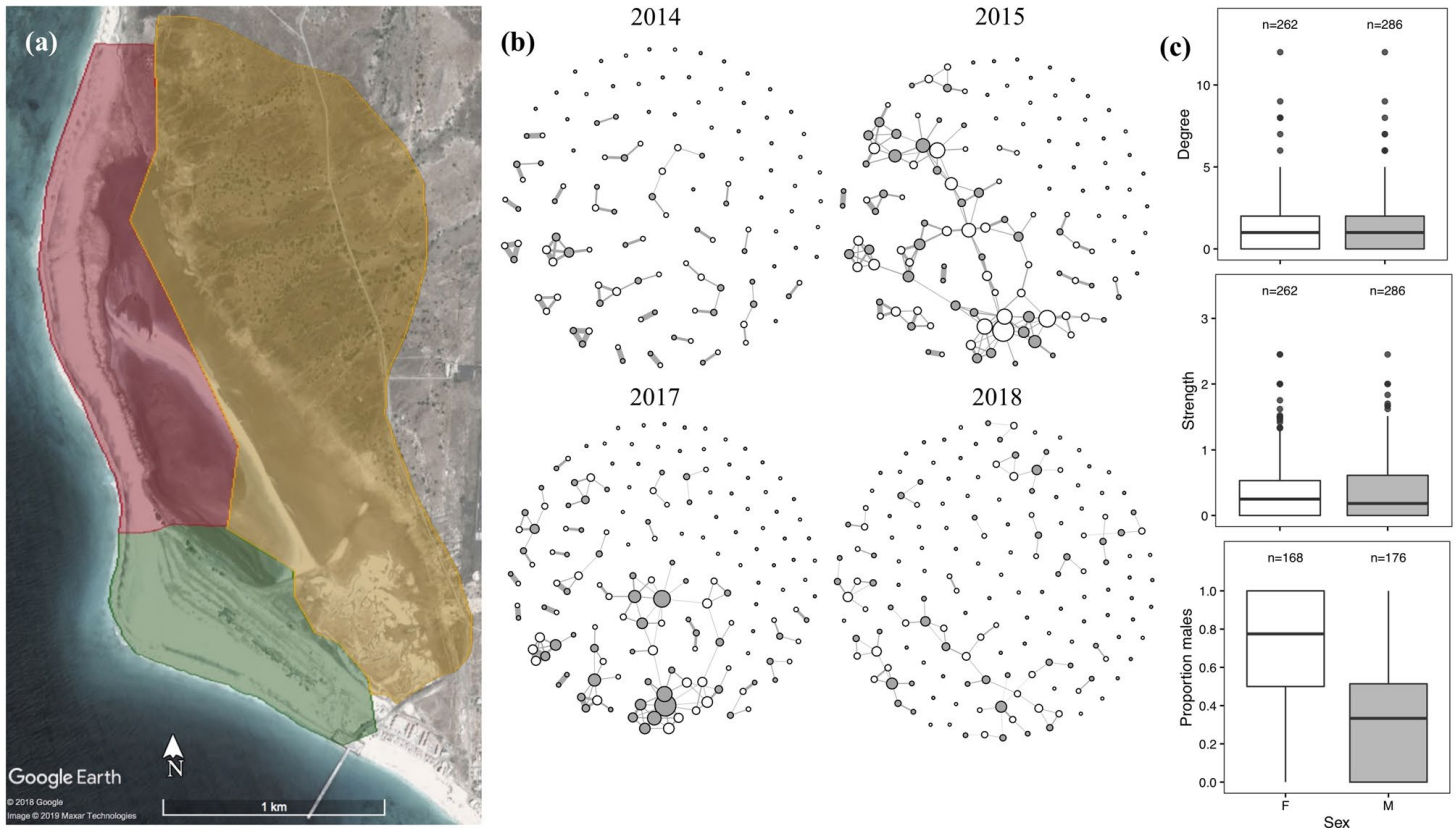
(**a**) Map of field site in Maio island, Republic of Cabo Verde. Coloured polygons highlight three different habitat regions including grassland (green), saltmarsh (red) and semi-desert (yellow). (**b**) Pre-breeding social networks of female (white) and male (grey) Kentish plover (*Charadrius alexandrinus*) from four years. Size of nodes indicates the number of social partners in the network (degree) and thickness of edges indicates the strength of social association between dyads. (**c**) Boxplots show the degree, strength and proportion of associates that were male for females (white) and males (grey) across all four pre-breeding social networks. Sample sizes are shown above boxplots. Map data: Google, Maxar Technologies.

We studied a population of Kentish plover on Maio island, Republic of Cabo Verde (Fig. 1a) between 2007 and 2018^51^. We categorised our study site into three areas encompassing three habitats that differ in their substrate and vegetation composition, “saltmarsh”, “grassland” and “semi-desert” (Fig. 1a, Supplementary Fig. S1, see Supplementary Information for details). For all years, we collected information on reproductive pairing, nest locations and nesting success using standardised methodologies^47,52^. Nesting attempts were considered successful if at least one egg of a clutch successfully hatched. We restricted analyses of breeding information to between August and December when the majority of breeding occurs. For a subset of years we conducted repeated surveys outside of the breeding season in two of the three habitats (saltmarsh and grassland, Fig. 1a, Supplementary Information Figs. S1, S2). During these non-breeding surveys we recorded the spatial locations of individual colour-ringed birds (approximately 80% of the adult breeding population is colour-ringed^51^, see Supplementary Information for details). The number of sightings and birds observed during non-breeding surveys across years are provided in Supplementary Information Table S1 and Fig. S3, S4.

### Network construction

Pre-breeding social networks were constructed from the recorded spatial associations of birds observed during non-breeding surveys in four years (see Supplementary Information Figs. S2–S4). Associations in social networks were inferred via the temporal and spatial proximity of individuals. Individuals that were located within a 10-min sampling period were assumed to be in association using a gambit of the group approach with a 20 m chain rule^53^ using package “spatsoc” version 0.1.9^54^. Edges between dyads were weighted by the simple ratio index (*SRI*)^17,55^ (see Supplementary Information for details). We utilise *SRI* as the weighted measure of dyadic association between individuals throughout analyses of social networks. To minimise the chances of including early breeding behaviour in pre-breeding social networks, we excluded non-breeding surveys sampled within 14 days of the start of each breeding season. For individuals we characterised their node degree (number of associates in the network) and node strength (sum of an individual’s edge weights)^55^.

Breeding networks for all years were constructed between nests that temporally overlapped (Supplementary Fig. S5). Nests were considered to temporally overlap if the period of nest incubation (i.e. date the clutch was laid until the date incubation ended) overlapped for at least 1 day (see Supplementary Information for details, Supplementary Fig. S6). We then calculated the pairwise Euclidean distance in metres between the recorded nest locations.

### General statistical approach

All analyses were conducted using R 3.6.1^56^. We account for repeated measures of individuals using mixed-effects models with the package “lme4” version 1.1-21^57^ throughout. Model residuals were explored for violations of assumptions. Models with binary response variables or proportion data utilised binomial error structures, models with node degree or nest counts use Poisson error structures and for continuous response variables Gaussian error structures with a log-link were used where appropriate. Throughout, we provide full model results including all explanatory variables, sample sizes and error structures in supplementary tables (Supplementary Tables S2–S9). Analyses using pre-breeding social networks are limited to those years with non-breeding survey data (Supplementary Fig. S4), whereas other analyses utilise available breeding data from all years (2007–2018; Supplementary Fig. S6).

Analyses of network data utilise randomisation tests where appropriate. Randomisation tests are used to assess whether observed relationships are more extreme than can be expected by chance due to random association patterns, while controlling for observed properties of the network e.g. sex ratio or the frequency in which individuals are sampled^17,55,58,59^. Randomisations of social network data were conducted using package “spatsoc” version 0.1.9 by randomising associations between individuals within any given 10-min sampling period of the pre-breeding surveys, thus following the original sampling pattern of the data to avoid generating biologically unrealistic randomised networks, as recently recommended^54^. Observed model estimates were then compared to the null distribution of effect sizes from models based on 10,000 randomisations of the social network data. We calculated two-tailed p-values based on the fraction of effects, including the observed effect, at least as extreme as the observed effect as outlined in Ruxton & Neuhäuser^60^.

In addition, we use randomisation tests to control for potential effects of site or habitat fidelity on patterns of reproductive pairing or nesting decisions. For example, if birds rarely move between habitats, site fidelity may drive correlations between sociality before breeding and reproductive pairing during breeding, simply because philopatric individuals mate locally. We account for this using custom habitat specific randomisation tests to ask if observed correlations are greater than expected by chance given complete habitat fidelity. For habitat specific randomisations where mating patterns were the variable of interest, the identity of individuals in mating pairs within a breeding season was randomized only among pairs that nested within the same habitat. Where nesting patterns was the variable of interest, the location and incubation period of nests within a breeding season were randomised only among pairs that nested in the same habitat (see Supplementary Information for details).

### Is pre-breeding social structure associated with pairing and nest success?

We first assessed whether there were any sex differences in sociality (Supplementary Table S2). Models included node degree, node strength or the proportion of individual’s associates in the network that were male, as response variables. Fixed effects included sex and year as a 4-level factor and individual identity as a random effect. We utilised randomisations to test whether any effect of sex was greater than expected under random patterns of social associations.

We then tested if pre-breeding social structure was related to the formation of breeding pairs. Models included a binary response variable indicating whether each male and female were part of a breeding pair (Supplementary Table S3). Fixed effects included the *SRI* between all possible pairs from pre-breeding social networks and year as a fixed 3-level factor. The identities of each individual in the dyad were included as crossed random effects. All possible combinations of males and females with both pre-breeding social and breeding information available for a given year were represented in the model. Due to the small number of breeding pairs in 2017, this year was not included in analyses (Supplementary Fig. S6). Importantly, individuals with more social connections in pre-breeding surveys are more likely to have associated with their future breeding partner at least once, compared to individuals with fewer social connections, simply because they have more associates. We therefore used randomisations as described above, that randomised the identity of individual’s mating partners. Randomisation tests therefore allow us to ask whether random patterns of pairing among breeders could generate a similar association between their observed pre-breeding sociality and pair formation. In addition, we explored whether habitat fidelity may impact on pair formation. To do so, we repeated the analyses above including only breeding pairs with recorded nest locations (Supplementary Table S3) and used habitat specific randomisations to test if any relationship between sociality and pair formation was greater than expected by chance given random patterns of pairing within local habitats. To confirm that any relationship between pre-breeding sociality and reproductive pairing was not driven by a subset of pairs that began breeding earlier than the defined breeding season, we repeated above analyses limiting pre-breeding social data to (i) exclude non-breeding survey data not for 14 but 30 days prior to the start of the breeding season or (ii) excluding non-breeding survey data for the month of May (when any breeding outside of the core breeding season, i.e. Aug–Dec, is most likely to occur). Results were qualitatively similar to those presented here (data not shown).

We then assessed whether pre-breeding social structure was related to nesting success. Models included a binary response indicating whether the pairs’ nesting attempt was successful. Fixed effects included the *SRI* between pairs from pre-breeding social networks, year as a 3-level factor and the identities of individuals in the pair as random effects. To confirm if nesting habitat was likely to impact on these results, we repeated this analysis for those nests for which habitat locations were available and included habitat as a 2-level factor. Due to the restricted number of pairs which had this additional nest location data, we used models without random effects and included only the first record for individuals which had multiple consecutive nesting attempts (Supplementary Table S4).

### Is pre-breeding social structure associated with breeding structure?

We assessed whether the overall sociality of individuals was related to the spatial and temporal organisation of nesting (i.e. breeding structure). Models assessing the relationship between temporal synchrony of breeding and overall sociality included the number of nests that overlapped with the nesting attempt of the focal bird as a response variable. Fixed effects included an individual’s node degree or node strength in pre-breeding social networks and year as a 3-level factor (Supplementary Table S5). Random effects included the identity of individuals and their nest. For models assessing the relationship between spatial organisation of nests and overall sociality we repeated the above models, replacing the response variable with the average distance between the nest of the focal bird and the nests of concurrent overlapping nests (Supplementary Table S5). Crucially, different habitats may differ in their physical configuration, density of nests and nest phenology. Therefore, we used randomisation tests, where nest locations and timing were randomised across breeding pairs within habitats (i.e. within saltmarsh or grassland). These randomisations allow us to explore whether observed relationships between sociality and temporal synchrony or spatial proximity were more than expected by chance given the local habitat in which individuals chose to nest.

We next asked whether pairwise pre-breeding social relationships between individuals was related to their breeding networks. We tested whether the pairwise strength of association (*SRI*) between dyads in the social network (excluding associations between breeding pairs) correlated with (i) the probability that those dyads incubated their nests at the same time and (ii) the Euclidean distance between their nests (Supplementary Table S6). Response variables were thus either (i) a binary variable indicating whether nests of dyads were concurrent (i.e. overlapped in their incubation period) or the Euclidean distance in metres between the nests of dyads. We included all focal nests from saltmarsh and grassland (i.e. habitats in which social network data was collected). Fixed effects included the habitat of focal nest as a 2-level factor and year as a 3-level factor. The identities of each individual in a given dyad and the identity of their nest were included as random effects. We then used habitat specific randomisation tests, where nest locations were randomised across breeding pairs within breeding habitat, to assess whether any correlation between *SRI* and response variables was significantly more extreme than expected from random selection of nest sites within a given habitat.

To examine whether breeding structure may determine nesting success, models included a binary response variable indicating whether the focal nest was successful (Supplementary Table S7). Fixed effects included the number of temporally overlapping nests, average Euclidean distance to overlapping nests, the Euclidean distance to nearest temporally overlapping nest, the habitat of the nest as a 3-level factor and a linear and quadratic term for the Julian laying date. Random effects included year. Julian date was variance standardised before analysis to avoid collinearity between linear and squared terms^61^. To explore the potential for collinearity between predictors in this model (e.g. number of overlapping nests and Julian date) we calculated variance inflation factors (VIFs) which revealed low potential for collinearity to influence our results (VIFs < 2).

Finally, temporally overlapping nests may not represent cases where breeding neighbours functionally interact if they are isolated by large distances. We therefore asked if the number of temporally overlapping nests within 100 m of a focal nest (an approximate distance under which parents likely interact) was associated with nesting success (Supplementary Table S7). The model included a binary response variable indicating nesting success. Fixed effects included the number of temporally overlapping nests within 100 m, habitat of the nest as a 3-level factor and a linear and quadratic term for Julian laying date. Random effects included year.

### Is breeding structure associated with future breeding decisions?

To explore how breeding social structure may influence future decisions over breeding dispersal we asked whether future nest locations used by individuals were related not only to the success of an individual’s own previous nesting attempt but also the nesting success of previous social neighbours.

We first focused on the effect of an individual’s own previous nesting success. For individuals who bred in at least two consecutive years we tested whether breeding dispersal for their subsequent nesting attempt in the following year was influenced by their nest success in the previous year (Supplementary Table S8). Models included a binary response variable of whether the individual remained in the same habitat or dispersed to another. Fixed effects included nest success and the habitat of the previous nesting attempt. Random effects included the identity of the focal bird and year. Furthermore, we repeated the above analysis using a finer resolution analysis, replacing the binary response variable, with the Euclidean distance between previous and future nesting attempt (Supplementary Table S8).

Finally, we assessed the effect of the nesting success of an individual’s local breeding neighbours in its previous nesting attempt on its future nest choice location. For individuals who bred in two consecutive years and had overlapping neighbouring nests within 100 m in the previous season, we tested whether the Euclidean distance between previous and future nesting attempt was associated with the fate of their previous nest and the proportion of overlapping neighbouring nests that were successful during the previous nesting attempt (Supplementary Table S9). The habitat of their previous nest was included as a fixed factor. Due to the low number of nests which had neighbours within 100 m and nest success data for neighbours, we used generalised linear models that included only the first record for individuals which had multiple consecutive nesting attempts.

## Results

### Is pre-breeding social structure associated with pairing and nest success?

Pre-breeding social networks were sparse (mean network density ± 1 SE = 0.011 ± 0.003 Fig. 1b), with few connections between individuals (grand mean degree ± 1 SE = 1.408 ± 0.360, Fig. 1b). In our observed pre-breeding social networks males and females had similar degree (degree, Fig. 1c), while in the randomised networks females tended to be more connected meaning the difference between males and females was significantly smaller than expected if birds were associating randomly, p^rand^ < 0.001, Supplementary Table S2). Overall node strength was not significantly associated with sex (strength; *p*^*rand*^ = 0.860, Fig. 1c, Supplementary Table S2). In addition, females associated proportionally more with males than expected by chance (*p*^*rand*^ = 0.004, Fig. 1c, Supplementary Table S2).

**Figure 2:**
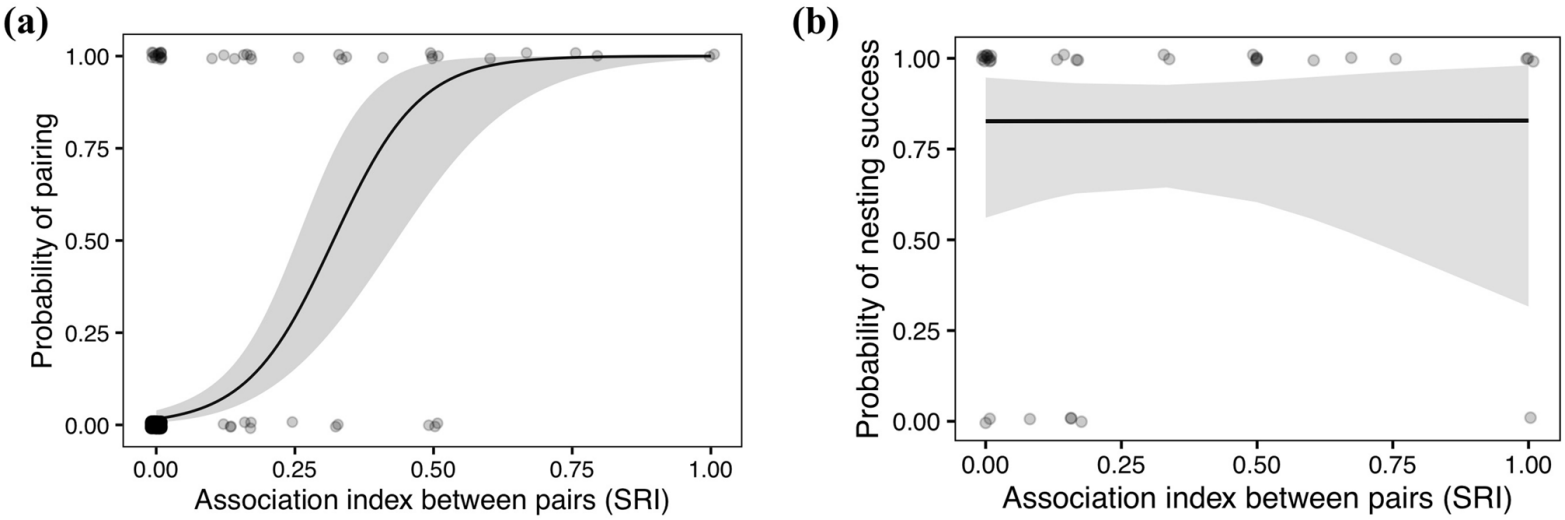
(**a**) The relationship between the index of social association (simple ratio index; *SRI*) between breeding pairs of male and female Kentish plover (*Charadrius alexandrinus*) in pre-breeding social networks and the probability that those individuals were a breeding pair during the subsequent breeding season. (**b**) The association index between breeding pairs of males and females (simple ratio index; *SRI*) in pre-breeding social networks and the probability that their subsequent nesting attempt was successful. Solid lines and shaded areas represent model predictions and 95% confidence intervals. Points are randomly shifted 0.01% vertically and 0.01% horizontally to minimise overplotting of points. The darkness of shaded points indicates the density of overlapping points.

The strength of social associations between male and female dyads in pre-breeding social networks (*SRI*) was positively associated with the probability that those individuals bred together as a pair in the subsequent breeding season (Fig. 2a, Supplementary Table S3). Of all 84 individuals included, 34 dyads had an *SRI* above zero, of which 22 were subsequently breeding pairs. Randomisation tests revealed that the observed relationship between social associations and pair formation was stronger than expected by chance compared to random pairing among breeding individuals (*p*^*rand*^ < 0.001). Similarly, randomisations that control for habitat fidelity revealed that individuals that socialise more bred together more than expected by chance in the non-breeding season (*p*^*rand*^ < 0.001, Supplementary Table S3).

The strength of association between individuals in pre-breeding social networks was not related to the nesting success of breeding pairs (Fig. 2b, Likelihood ratio test, LRT ${\chi }_{1}^{2}$= 0.000, p = 0.994, Supplementary Table S4).

### Is pre-breeding social structure associated with nesting structure?

The overall number of pre-breeding social associates (node degree) or the overall node strength of an individual was not related to the distance between their nest and other concurrently incubated nests (degree *p*^*rand*^ = 0.243; strength *p*^*rand*^ = 0.312, Supplementary Table S5) or the number of concurrently incubated nests (degree *p*^*rand*^ = 0.933; strength *p*^*rand*^ = 0.613, Supplementary Table S5).

At the level of individual dyads, the strength of associations in pre-breeding social networks was negatively related to the distance between the nests of those dyads (i.e. individuals who associate more strongly in the social network nested closer together, Supplementary Table S6), however, randomisation tests revealed this relationship was not stronger than expected compared to random nesting patterns after controlling habitat fidelity (*p*^*rand*^ = 0.311). In addition, the association between dyads in social networks was not linked to the probability that the incubation period of dyads overlapped (Supplementary Table S6, *p*^*rand*^ = 0.567).

The number of temporally overlapping nests was significantly and positively related to nesting success (LRT ${\chi }_{1}^{2}$ = 10.237, p = 0.001, Supplementary Table S7). However, the average Euclidean distance to temporally overlapping nests and the Euclidean distance to the nearest overlapping nest was not associated with nesting success (average distance: LRT ${\chi }_{1}^{2}$ = 1.687, p = 0.194, minimum distance: LRT ${\chi }_{1}^{2}$ = 1.766, p = 0.184, Supplementary Table S7). The habitat, and Julian date on which a nest was laid, was not significantly related to nesting success (LRT habitat: ${\chi }_{2}^{2}$ = 3.972, p = 0.137, Julian date: ${\chi }_{1}^{2}$ = 0.267, p = 0.605, Julian date^2^: ${\chi }_{1}^{2}$ = 1.660, p = 0.198, Supplementary Table S7). Similarly, the probability that a nesting attempt was successful was not associated with the number of nests within a 100 m radius (LRT, ${\chi }_{1}^{2}$ = 1.001, p = 0.317, Supplementary Table S7).

### Is nesting structure associated with future nest choice?

**Figure 3:**
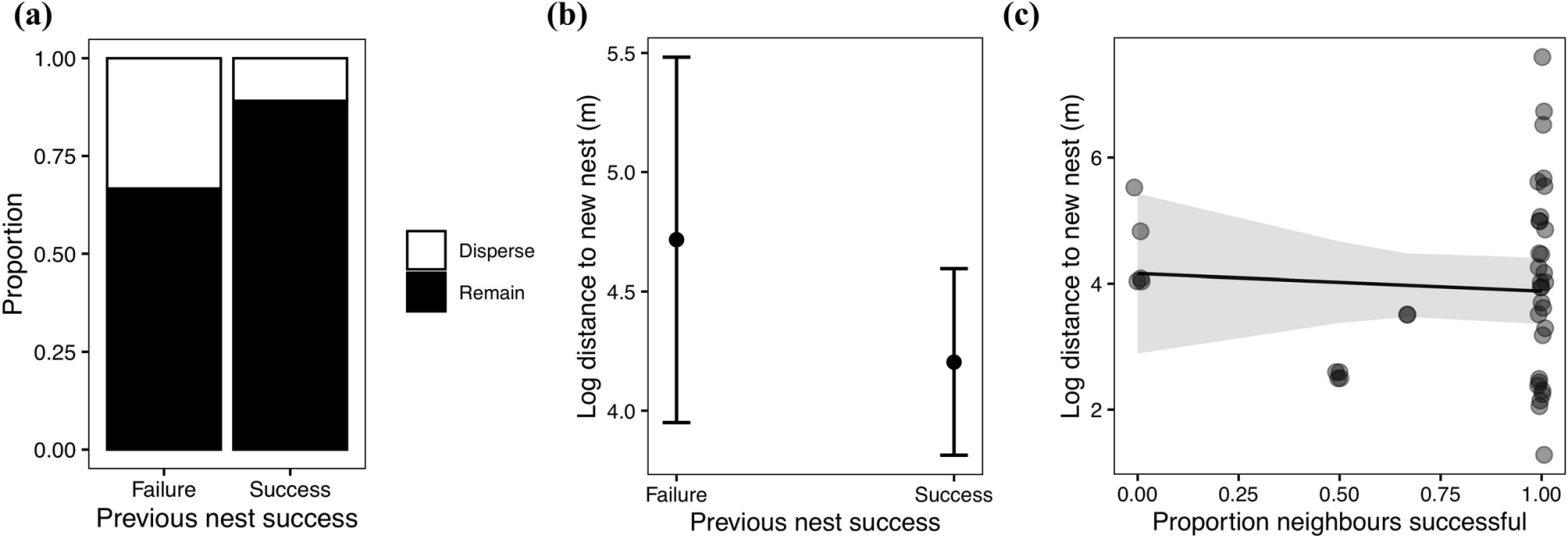
(**a**) The proportion of individuals who remain in the same habitat (black) or disperse (white) for their subsequent nesting attempt the following year as a function of the success of their nesting attempt in the previous year. (**b**) The relationship between the success of an individual’s previous nesting attempt and the distance in metres to the location of their subsequent nesting attempt the following year. Points show predicted means and error bars represent 95% confidence intervals. (**c**) The relationship between proportion of temporally overlapping neighbouring nesting attempts that were successful in the previous nesting attempt and the distance in metres to the location of the subsequent nesting attempt the following year. Line shows model predictions and shaded areas show 95% confidence intervals.

Individuals successful in their previous nesting attempt were more likely to remain in the same habitat for their subsequent attempt with 10 of 15 (66.67%) birds that failed in their previous attempt remaining while 82 of 92 (89.13%) birds successful in their previous attempt remaining (LRT ${\chi }_{1}^{2}$= 8.485, p = 0.004, Fig. 3a, Supplementary Table S8). However, this analysis was limited by variation in dispersal, as the majority of individuals tended to remain in the same breeding habitat for subsequent nesting attempts in the following year across all habitats (remaining/total: grassland = 40/47, saltmarsh = 34/37, semi-desert = 18/23). Individuals whose previous nesting attempt was successful did not nest significantly closer to their previous nest location for future attempts (LRT ${\chi }_{1}^{2}$= 0.7114, p = 0.399, Fig. 3b, Supplementary Table S8).

The distance between previous and future nesting attempts was not significantly related to the proportion of overlapping neighbouring nests within 100 m that were successful (LRT ${\chi }_{1}^{2}$ = 2.005, p = 0.157, Fig. 3c, Supplementary Table S9).

## Discussion

It is becoming increasingly appreciated that the social environment has key repercussions for patterns of temporal and spatial breeding distribution and success^12,28,38,39,41,44^. However, studies rarely examine the role of social structure across different aspects of reproduction together in one system, thus the relative influence of social structure across different components of reproduction, from mating, to reproductive success and future breeding dispersal are hard to determine. Here we utilised information on pre-breeding sociality in the ground-nesting shorebird, the Kentish plover, in combination with information on mating decisions, nest location and breeding success, to explore the impact of social structure on breeding ecology across multiple stages of reproduction. Sociality before breeding was connected with patterns of reproductive pairing in the subsequent breeding season. However, at the landscape scale habitat fidelity and personal breeding experience was associated with the spatial organisation of breeding pairs.

Our results show that males and females who associate more strongly together outside of the breeding season are more likely to form reproductive pairs. Randomisation tests controlling for habitat fidelity yielded similar results, suggesting that restricted movement of individuals within habitats alone cannot explain the relationship between social associations and the likelihood of breeding as a pair. While it is possible that local site fidelity may occur at even more restricted spatial scales than those taken into account in habitat specific randomisations, previous works assessing movements of individual Kentish plover within and between breeding seasons suggest birds can regularly travel distances exceeding those captured in our study site^50,62,63^. In addition, social networks displayed more male–female associations and fewer male-male associations than was expected from our network randomisations. This indicates that the observed frequencies of male-male and male–female interactions cannot be explained by random patterns of social grouping, and suggests a role for non-random social processes in generating the frequency of intra- and intersexual interactions. One limitation of the observational approach used to capture social associations is a relatively low mean number of repeat sightings per individual compared to automated approaches to gather social network associations^55^. However, despite these restrictions our results identify pre-breeding sociality as a potentially strong predictor of future pair formation. Social behaviour before breeding may represent social strategies to find mates and/or facilitate bonding between breeding pairs. In great tits (*Parus major*), males with higher levels of activity and exploration, met their future breeding partners earlier and displayed stronger social affiliations with their future partners than less active and explorative males^28^. Longer-term social associations between future breeding pairs may also facilitate opportunistic breeding strategies. Prolonged associations between pairs may facilitate swift commencement of breeding if local environmental conditions conducive to breeding are unpredictable and subject to rapid change. Such strategies may be particularly relevant to Kentish plover on Maio where the onset of seasonal precipitation is expected to be related to rapid increases in food availability and breeding activity^64^. Future research should seek to understand in more detail the social behaviours between future pairs outside of the breeding season and the causes of variation in the social connectedness of future partners.

Socialisation between reproductive pairs before breeding may impact on the strength of pair-bonding and subsequent breeding success, for example if stronger pair-bonds increase the coordination of breeding behaviour between partners^28,37^. However, we found no evidence that the relative strength of pre-breeding social association between pairs impacted subsequent nesting success. The lack of relationship between social bonds and nesting success may result if any increase in the strength of pair-bonding only influences reproductive success at later stages of brood care (e.g. via chick development or chick survival) or if hatching success due to optimised coordination of incubation is limited compared to variation caused by predation. Nest predation is a principal cause of nest failure in shorebirds^5,65^ and therefore improvements in hatching success due to stronger pair bonds may be much harder to detect, requiring more intense sampling or only be evident under situations with lower nest predation or more extreme climactic conditions that increase the importance of efficient incubation behaviour^5,47^. Alternatively, increases in pair familiarity that impact on breeding success may operate over longer timescales than those observed here. Kentish plover pairs may divorce and remate within the same season or breed together over multiple years^48^. Therefore variation in familiarity developed over multiple breeding attempts may be more important, or instead determined more strongly by individual’s own breeding experience than the strength of pair bonds^37,66^.

After forming reproductive pairs, a key next stage in breeding is to locate an appropriate breeding or nesting location. The social environment during breeding may have key implications for individual fitness. For example, in Seychelles warblers (*Acrocephalus sechellensis*), increased familiarity between neighbouring territorial males reduces the frequency of physical fights between them and is associated with increased body weight and a decrease in indicators of physiological damage compared to unfamiliar neighbours^39^. Social structure is therefore predicted to influence the choice of breeding locations. Our results indicate that individuals who associate more before breeding also breed closer together. However, randomisation tests controlling for habitat fidelity suggest the main driver of nest proximity was the habitat choice of individuals. This is in contrast to more recent work in other species indicating a key role for social behaviour in the temporal and spatial organisation of breeding populations^28,41,67^. In wild zebra finches (*Taeniopygia guttata*), birds who breed in the same colony, and at the same time, have stronger social ties after reproduction, suggesting a strong relationship between social structure before and during breeding. Our results instead suggest that a combination of individual habitat preferences or site fidelity may predict the structure of breeding territories. Habitat preferences may be linked to local variation in ambient temperatures, predator densities, as well as the availability of different nest substrates or nest cover, which can have strong implications for the success of nesting attempts in Kentish plover via heat stress to eggs and predation risk^47,68–71^. In addition, we found no evidence that the number of breeding pairs within 100 m of a focal bird’s nest, influenced their nesting success. This suggests a limited role of local neighbours in shaping the breeding success of focal individuals. However, the total number of temporally overlapping nests at the level of the population was positively associated with nest success. This suggests the positive relationship between breeding synchrony and nesting success may reflect a temporal peak in the quality of ecological conditions under which most individuals commence a breeding attempt, such as food abundance, ambient temperatures or may indicate lower predation risk of individual nests associated with a dilution effect present at a scale greater than 100 m. On the other hand, the traits of local neighbours, such as aggressiveness, may play a greater role in determining breeding success than local densities alone. Furthermore, effects of between-breeder conflict on reproductive success may be limited to brood care stages where adult plovers may kill the chicks of other parents^72^.

Our results also shed light on the relative role of sociality and local ecology in shaping the genetic structure of populations via breeding dispersal. We show that individuals demonstrate strong habitat fidelity between nesting attempts across consecutive years. In addition, those individuals successful in their previous nesting attempt were more likely to remain in the same habitat in their subsequent nesting attempt the following year. Specifically, individuals’ previous breeding experience was associated with the choice of their future breeding site but not the distance from their previous to their future nest. This suggests that breeding dispersal is more strongly determined by habitat type than limited movement in general, which is in line with the relatively large possible dispersal distances previously reported for Kentish plover^50^. We found no evidence that the nesting success of local neighbours predicted the location of future nesting attempts. It may be that individuals do not collect or use social information on the nesting success of local neighbours, or the use of social information may be limited to only a subset of individuals. For example, in great tits, the use of conspecific information in nest choice is limited to pairs including older males^73^. Alternatively, the number of individuals within 100 m may not adequately capture the scale at which social information is collected. The use of social information in determining breeding site choice may be more pertinent to more colonially nesting species where the success of neighbouring individuals may be more readily accessed^42,44,74^, although there is evidence for the use of social information also in some non-colonial species^75^.

In summary, our results provide evidence that—at the landscape scale—site fidelity, habitat preferences and an individual’s previous breeding experience may be relatively stronger drivers of the structure of breeding territories and breeding dispersal than social structure. However, at more localised scales, differential social interactions throughout the non-breeding season may influence patterns of reproductive pairing. Manipulative studies of pre-breeding social behaviour, in combination with methods allowing more intensive behavioural resampling of individuals may provide more precise estimates of social structure and useful routes to determine the functional role of non-breeding social associations. In addition, comparative studies of non-breeding social structure across populations that vary in adult sex ratio and sex roles may provide insight into the role of sociality^33^. Finally, the link between pre-breeding sociality and mate choice suggests that processes that affect social structure before breeding may in turn impact on subsequent patterns of pairing. Future research should seek to explore generality of the potential for non-breeding sociality to carry over to mating system dynamics including patterns of mate choice and pair-bonding.

## Supplementary information

Supplementary Figures

Supplementary Information

Supplementary Data 1

Supplementary Data 2

Supplementary Data 3

## Data Availability

Data is available as supplementary material.
