# Supplementary figures and images for: The impact of social structure on breeding strategies in an island bird
Source: Sci Rep. 2020 Aug 17;10:13872. doi: 10.1038/s41598-020-70595-w (PMC7431420; doi:10.1038/s41598-020-70595-w)

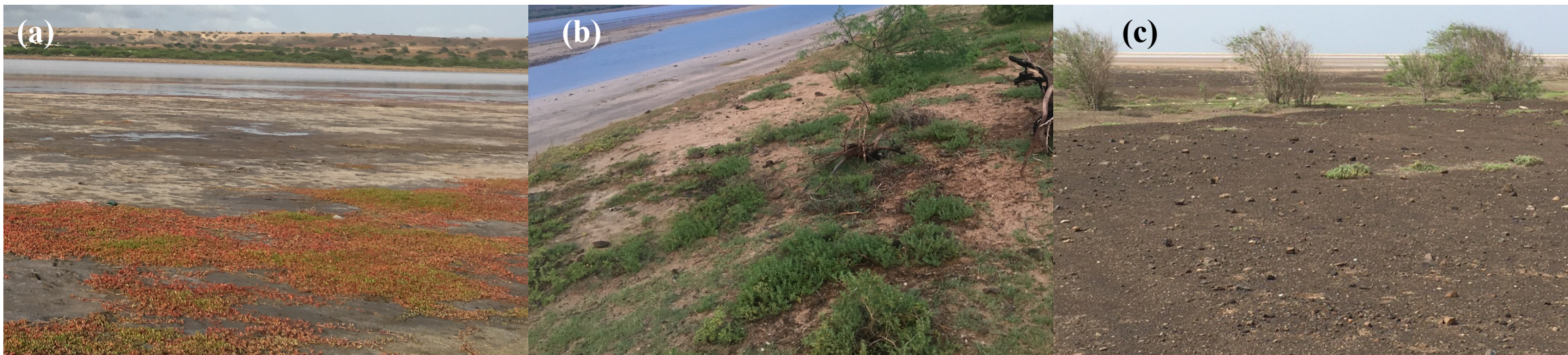

**Fig. S1**

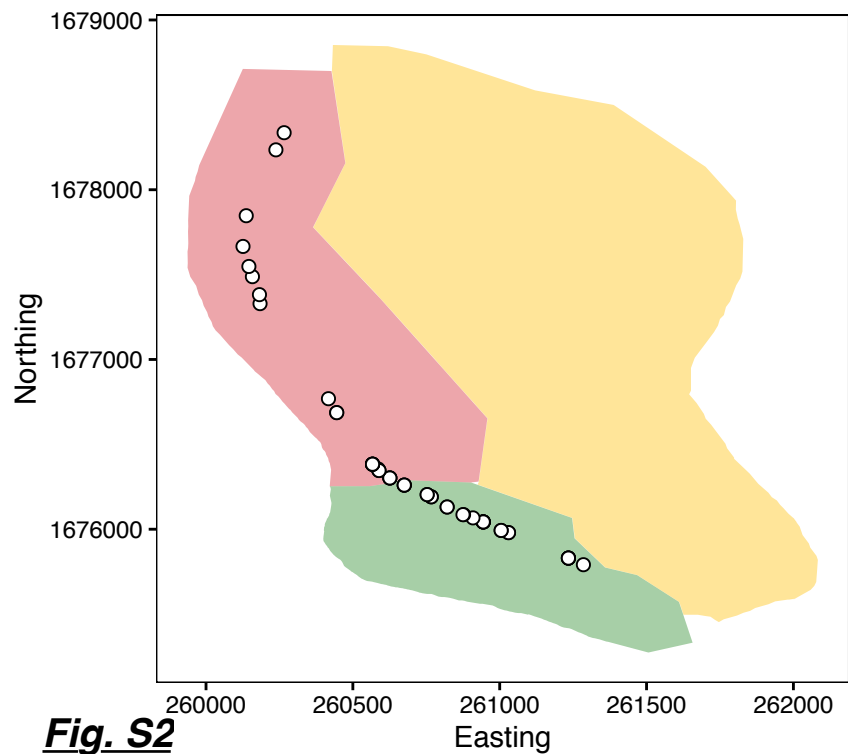

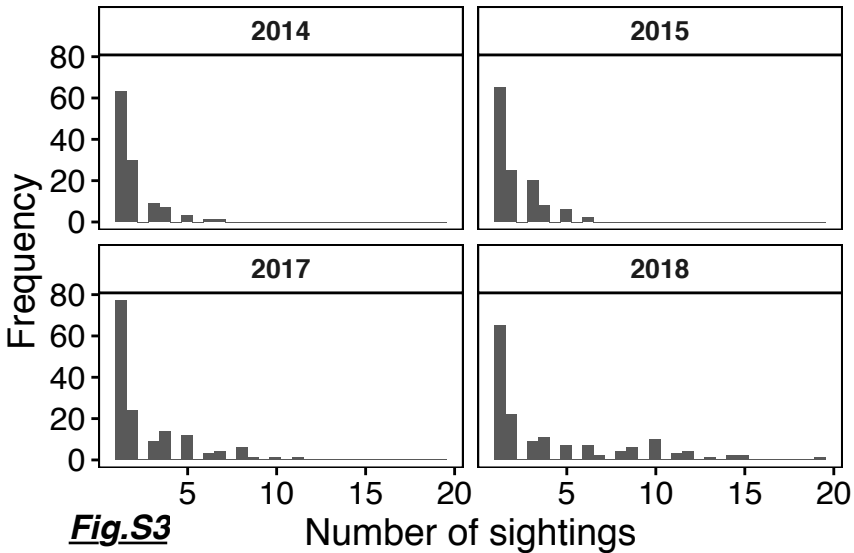

**Fig.S3**

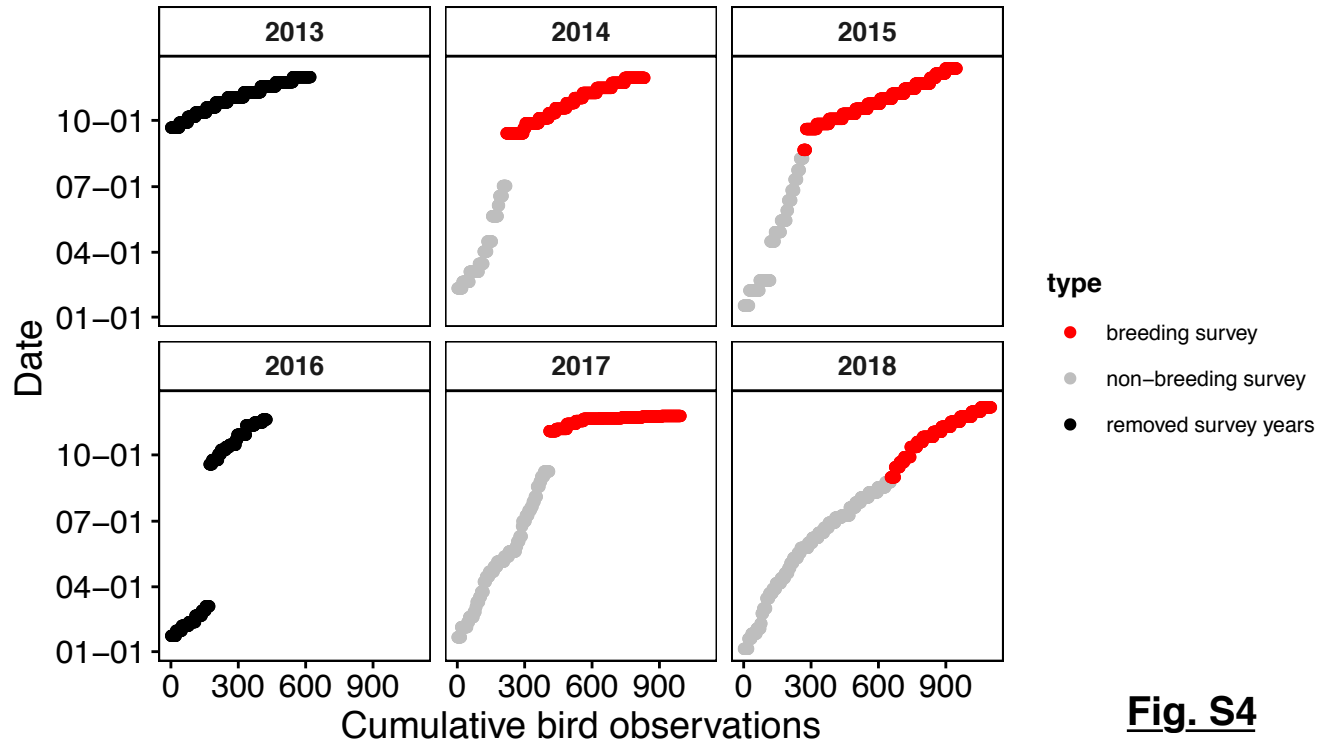

**Fig. S4**

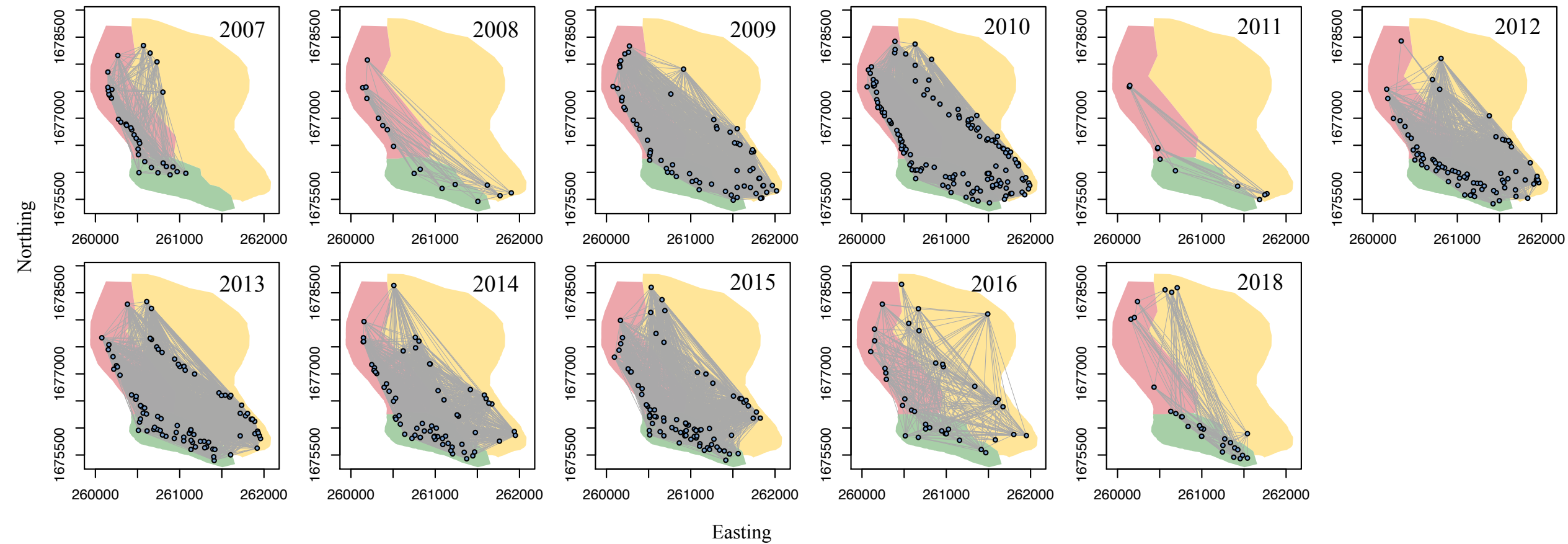

**Fig. S5**

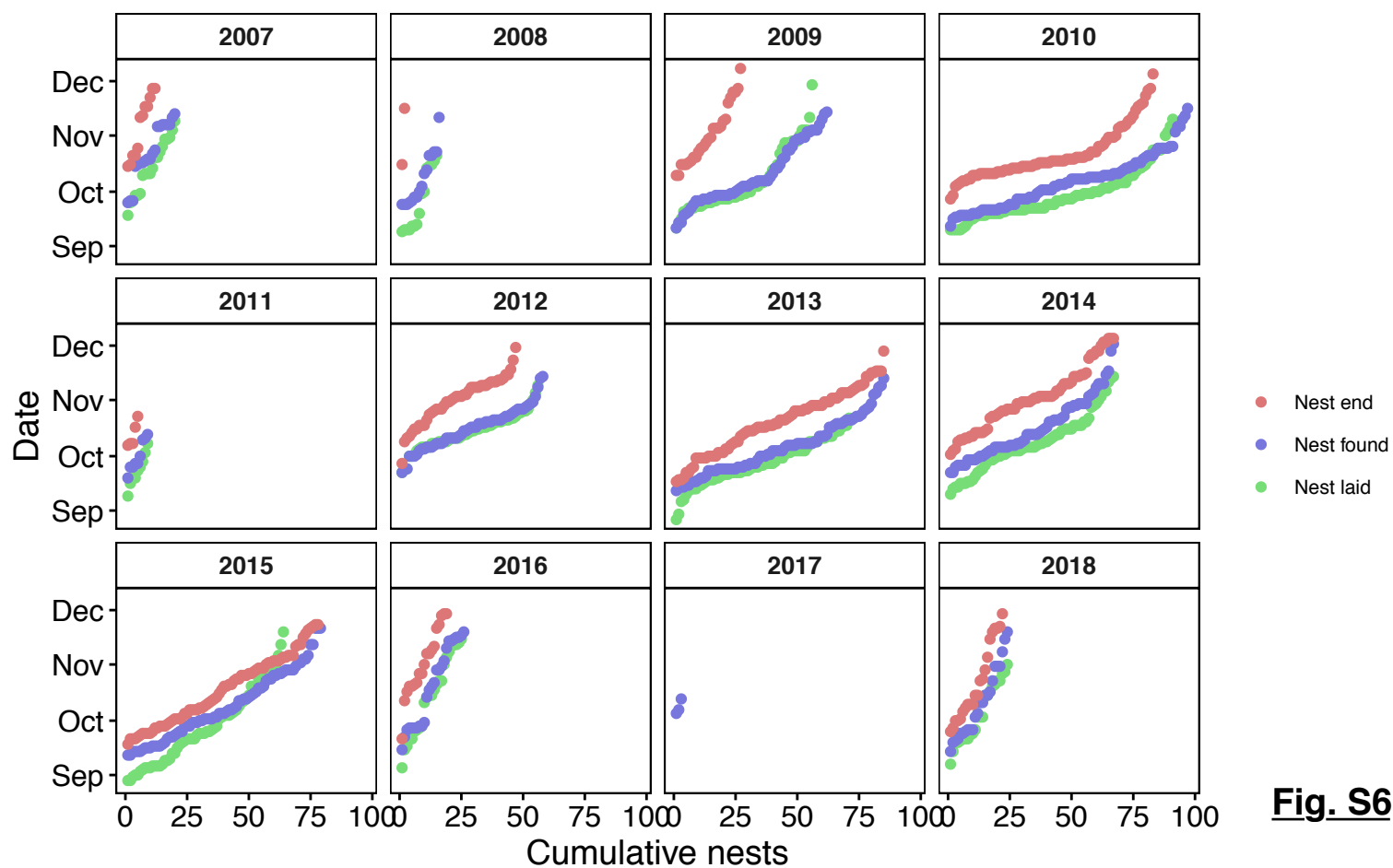

**Fig. S6**

Supplement: Supplementary file 1 — Supplementary Figures [file 41598_2020_70595_MOESM1_ESM.pdf]
