# Supplementary material for: The impact of social structure on breeding strategies in an island bird
Source: Sci Rep. 2020 Aug 17;10:13872. doi: 10.1038/s41598-020-70595-w (PMC7431420; doi:10.1038/s41598-020-70595-w)
Supplement: Supplementary file 2 — Supplementary Information [file 41598_2020_70595_MOESM2_ESM.docx]

Supplementary information for:

**The impact of social structure on breeding strategies in an island bird**

Grant C. McDonald^1,2*^, Noémie Engel^3^, Sara S. Ratão^4^ Tamás Székely^3,4,5^ and András Kosztolányi^1^

^1^Department of Ecology, University of Veterinary Medicine Budapest, Budapest, Hungary

^2^Edward Grey Institute, Department of Zoology, University of Oxford, Oxford, UK

^3^Milner Centre for Evolution, Department of Biology and Biochemistry, University of Bath, Bath, UK

^4^ FMB, Fundação Maio Biodiversidade, Cidade do Porto Inglês, Maio Island 6110, Cabo Verde

^5^ Department of Evolutionary Zoology and Human Biology, University of Debrecen, Debrecen, Hungary

**Supplementary methods**

***Field site***

### Field studies were conducted in the Salinas do Porto Inglês in the southwest of the island of Maio, Cabo Verde between 15°08' - 15°10'N and 23° 14- 23° 12'E (Carmona-Isunza et al. 2015). The site is a Ramsar listed wetland (Ramsar 1971; RIS 2013), containing diverse habitats characterised by differences in substrate and vegetation. We therefore categorised our field site into three broad habitats reflecting the key differences in substrate and vegetation including “Saltmarsh”, “Grassland” and “Semi-desert” (Fig. 1a). Saltmarsh habitat is characterised by a sandy substrate dominated by sesuvium plants (*Sesuvium portulacastrum*; Fig. S1a). Grassland habitat constitutes a grassy plateau composed of mainly short grass, small shrubs and a sandy substrate (Fig. S1b). The semi-desert habitat is characterised by dark rocky volcanic substrate punctuated by acacia trees (Prosopis jullflora) (Fig. S1c).

***Non-breeding survey data***

Survey data were collected all year round along a single transect approximately running from the southern end of the grassland habitat to the north of the saltmarsh habitat from a vehicle using binoculars and/or scope (Fig. S2). The number of repeated transects in each year is given in Table S1. For each repeated survey, observers travelled along the transect in a vehicle and stopped at a sampling point approximately every 100 metres. At each sampling point observers recorded the UTM coordinates of the sampling point and identities of birds observed via colour ring combinations. If a bird was observed between any 100 metre designated sampling point, observers also stopped and recorded the identities of birds as earlier described and this was also treated as a sampling point. For each bird, observers recorded the distance and direction of the bird from the observer. The distance and direction were then used in combination with observer UTM coordinates to estimate the location of each colour-ringed individual. Associations in pre-breeding social networks were then inferred from this non-breeding survey data using a system of temporal and spatial proximity, where birds recorded within the first 10 minutes of arriving at a sampling point were designated as being in association following the gambit of the group approach with a 20 metre chain rule (Franks *et al*. 2010) using package “spatsoc” version 0.1.9 (Robitaille *et al*. 2018). Edge weights between individuals in networks were calculated using the simple ratio index ($SRI=x/\left( ya+yb+yab+x \right)$ where *x* is the number of sampling periods individual *a* and *b* were observed together, *ya* and *yb* are the number of sampling periods *a* or *b* were observed in isolation and *yab* is the number of sampling periods both *a* and *b* were observed but were not in association (Krause et al. 2009). To minimise chances of including early breeding behaviour in our non-breeding data, we did not use information on social associations from surveys sampled within 14 days of the first nest being found or, when possible, the date the nest was first estimated to have been laid (Fig. S2-S4, lengthening the period between non-breeding and breeding season to 30 days before the first nest or excluding the month of May, when any breeding outside of the core breeding season is most likely, provided qualitatively similar results, data not shown).

***Breeding data***

### For breeding pairs identified at nesting stage, we recorded the UTM coordinates of nests, allowing the specific habitat (grassland, saltmarsh or semi-desert) and relative nest locations to be determined (Fig. S4). A subset of breeding pairs was identified only after a nesting attempt had been successful (i.e. during brood care). For breeding pairs identified only during brood care, the exact locations of their nesting attempt were unknown. For these pairs, it was recorded whether pairs were found either in semi-desert or non-semi-desert. For those analyses that use non-breeding social network data (i.e. collected only in saltmarsh or grassland) but do not require identification of specific habitat or exact nest location, we use all information available on breeding pairs identified at the nesting stage and brood care stage located in non-semi-desert. Whereas, for analyses that require information on breeding habitat or nest location (i.e. habitat specific randomisations) we restrict analysis to those pairs identified at the nesting stage.

To characterise breeding structure, we calculated within each year whether nests temporally overlapped in their period of incubation. Nests were considered to overlap (i.e. were concurrently incubated) if the period of incubation for both nests overlapped in time for at least 1 day. We calculated the date nests were laid by estimating the number of days that eggs had been incubated in newly found nests by floating the eggs in lukewarm water (Székely *et al*. 2008). Incubation end dates were estimated by visiting nests at four to five‐day intervals until approximately 20 days after which nests were visited daily (Székely *et al*. 2008; Kosztolányi *et al*. 2009). In a minority of cases, nests had incomplete laying date or completion dates. In these cases, nests were assumed to be incubated for 25 days. Nests for which no timing information was available and overlaps could not be identified were excluded.

When breeding data (e.g. pair formation or nesting patterns) were the variable of interest in statistical models we use randomisation tests. Randomisations tests allow us to ask if observed relationships are more extreme than can be expected by chance due to random mating or nesting patterns. These randomisations operate by randomly varying the identity of individual’s mating partners or the nest identity of pairs. Randomisation tests therefore allow us to ask whether random patterns of pairing or nesting among breeders could generate a similar association between e.g. observed pre-breeding sociality and pair formation. We also use randomisation tests to control for potential effects of site or habitat fidelity on patterns of reproductive pairing or nesting decisions. The habitat specific randomisations followed the same logic as described above but limited the randomisation of the identity of mating partners or the nest identity of pairs to operate only within a given habitat category (i.e. saltmarsh or grassland). This approach is useful if for example, if birds rarely move between habitats both before and during breeding. In such cases, habitat fidelity may drive a correlation between pre-breeding sociality and pair formation at breeding, largely because individuals choose to mate local within their local habitat. By using habitat specific randomisations we are able to test if observed correlations are greater than expected by chance given complete habitat fidelity.

**Supplementary figure legends**

**Figure S1** Photographs show representative habitat characteristics from (a) saltmarsh (b) grassland and (c) semi-desert in Maio, Cabo Verde.

**Figure S2** Plot shows locations of observations of Kentish plover (*Charadrius alexandrinus*) along an example survey transect from 2015. Colours highlight different habitats including saltmarsh (red), grassland (green), semi-desert (yellow).

**Figure S3** Distribution of sightings per individual Kentish plover (Charadrius alexandrinus) for non-breeding survey data collected across four years (2014, 2015, 2017 and 2018).

**Figure S4** Cumulative count of observations from survey data collected in the saltmarsh and grassland habitats. Black points represent years not included in analyses due to limited survey data throughout the non-breeding season. Red points represent data not included in pre-breeding social networks because survey data falls during the breeding season or within 14 days before the beginning of the breeding season. Grey points represent observations available in non-breeding survey data (up to 14 days before the first nest of the breeding season was laid/identified) used for the construction of pre-breeding social networks.

**Figure S5** Locations of Kentish plover (Charadrius alexandrinus) nests available from breeding seasons between 2007-2018. Axes show universal transverse mercator coordinates. Connections between nests indicate temporal overlap in their incubation period. Coloured polygons highlight three different habitat regions including grassland (green), saltmarsh (red) and semi-desert (yellow).

**Figure S6** Cumulative count of nests for breeding seasons 2007-2018 and the dates in which nests were laid (Nest laid), found (Nest found) and on which incubation ended (Nest end). Nests with no date information are not represented. Nests not laid between August and December (i.e. core breeding season) are excluded.

**Supplementary tables**

| **Table S1.** Summary of non-breeding survey data collected across four years showing all observations of individuals observed within 10 minutes of each sampling point along survey transects. | | | | |
| --- | --- | --- | --- | --- |
| **Year** | **No. sightings** | **No. transects** | **No. individuals** | **Mean sightings per individual** |
| 2014 | 206 | 10 | 114 | 1.81 |
| 2015 | 256 | 12 | 126 | 2.03 |
| 2017 | 402 | 30 | 152 | 2.64 |
| 2018 | 644 | 31 | 156 | 4.13 |

| **Table S2.** Relationship between social network metrics and sex. Model for degree used Poisson error structures, node strength used Gaussian error structures with a log-link and proportion males used binomial error structures. Models included data from four years (2014, 2015, 2017 and 2018). Results are presented as treatment contrasts with respect to the intercept. *z*-values are given for node degree and proportion male associates models, and *t*-values for node strength model. | | | | | | |  |
| --- | --- | --- | --- | --- | --- | --- | --- |
| **Response** | **Fixed effects** | **Estimate** | **Standard Error** | ***z/t*-value** | **No. individuals/**  **observations** | **Random effects** | |
| Node degree | Intercept (Female, 2014) | -0.302 | 0.126 | -2.398 | 269/548 | Individual identity | |
|  | Sex (Male) | -0.033 | 0.107 | -0.312 |  |  | |
|  | 2015 | 0.92 | 0.121 | 7.598 |  |  | |
|  | 2017 | 0.597 | 0.123 | 4.838 |  |  | |
|  | 2018 | 0.033 | 0.136 | 0.243 |  |  | |
| Node strength | Intercept (Female, 2014) | 0.342 | 0.039 | 8.776 | 269/548 | Individual identity | |
|  | Sex (Male) | -0.025 | 0.043 | -0.568 |  |  | |
|  | 2015 | 0.144 | 0.033 | 4.373 |  |  | |
|  | 2017 | -0.019 | 0.033 | -0.566 |  |  | |
|  | 2018 | -0.283 | 0.037 | -7.618 |  |  | |
| Proportion male associates | Intercept (Female, 2014) | 0.362 | 0.215 | 1.688 | 269/548 | Individual identity | |
|  | Sex (Male) | -1.033 | 0.154 | -6.718 |  |  | |
|  | 2015 | -0.098 | 0.238 | -0.411 |  |  | |
|  | 2017 | 0.697 | 0.245 | 2.846 |  |  | |
|  | 2018 | 0.307 | 0.268 | 1.146 |  |  | |

| **Table S3.** Relationship between the weight of individual pairwise social associations (*SRI*) and the probability dyads form a breeding pair (Paired). Models included data from three years (2014, 2015 and 2018). Models used binomial error structures. Results are presented as treatment contrasts with respect to the intercept. | | | | | | | |
| --- | --- | --- | --- | --- | --- | --- | --- |
| **Response** | **Fixed effects** | **Estimate** | **Standard Error** | ***z*-value** | **No. individuals/**  **observations** | **Random effects** |  |
| Paired (0,1) | Intercept (2014) | -3.768 | 0.359 | -10.503 | 84/1090 | Male identity, female identity |  |
|  | Simple ratio index (*SRI*) | 12.666 | 1.749 | 7.241 |  |  |  |
|  | 2015 | -0.457 | 0.465 | -0.984 |  |  |  |
|  | 2018 | 0.771 | 0.633 | 1.218 |  |  |  |
| Paired (0,1)  (Saltmarsh/Grassland only) | Intercept (2014) | -4.719 | 0.907 | -4.610 | 64/624 | Male identity, female identity |  |
|  | Simple ratio index (*SRI*) | 14.021 | 3.611 | 3.883 |  |  |  |
|  | 2015 | -0.216 | 0.617 | -0.350 |  |  |  |
|  | 2018 | 0.830 | 0.854 | 0.971 |  |  |  |

**Table S4.** Model results assessing the relationship between the weight of individual pairwise social associations (*SRI*) and the probability that a pairs nesting attempt was successful (Nest Success). Models included data from three years (2014, 2015 and 2018). Models used binomial error structures. Results are presented as treatment contrasts with respect to the intercept. For our model controlling for habitat (Grassland. Saltmarsh) we include only the first recorded nesting attempt for individuals that had more than one nesting attempt.

|  |  |  |  |  |  |  |
| --- | --- | --- | --- | --- | --- | --- |
| **Response** | **Main effects** | **Estimate** | **Standard Error** | ***z*-value** | **No. nests** | **Random effects** |
|  |  |  |  |  |  |  |
| Nest Success(0,1) | Intercept (2014) | 1.941 | 0.945 | 2.055 | 35 | Male identity. Female identity |
|  | Simple ratio index (*SRI*) | 0.012 | 1.548 | 0.008 |  |  |
|  | 2015 | -0.073 | 1.082 | -0.067 |  |  |
|  | 2018 | -3.041 | 1.438 | 2.116 |  |  |
| Nest Success(0,1) | Intercept (Grassland, 2014) | 3.011 | 1.799 | 1.674 | 14 | - |
|  | Simple ratio index (*SRI*) | -1.031 | 1.979 | -0.521 |  |  |
|  | 2015 | -1.621 | 1.485 | -1.092 |  |  |
|  | Saltmarsh | -1.079 | 1.494 | -0.722 |  |  |

| **Table S5.** Relationship between the number of temporally overlapping nests and the mean Euclidean distance in metres to overlapping nests with individual node degree and strength. Degree and strength are scaled to have a mean of zero and a standard deviation of 1. Models included data from three years (2014, 2015 and 2018). Models for number of overlapping nests used Poisson error structures and mean Euclidean distances used Gaussian error structures where mean Euclidean distances were square-root transformed. Results are presented as treatment contrasts with respect to the intercept. *z*-values are given for models of no. overlapping nests and *t*-values are given for mean Euclidean distance models. | | | | | | |  |
| --- | --- | --- | --- | --- | --- | --- | --- |
| **Response** | **Fixed effects** | **Estimate** | **Standard Error** | ***z/t*-value** | **No. individuals/nests/**  **observations** | **Random effects** |  |
| No. overlapping nests | Intercept (2014) | 3.842 | 0.055 | 69.466 | 82/95/123 | Individual identity, Nest identity |  |
|  | Degree | -0.003 | 0.025 | -0.116 |  |  |  |
|  | 2015 | -0.178 | 0.074 | -2.416 |  |  |  |
|  | 2018 | -1.287 | 0.11 | -11.649 |  |  |  |
| Mean Euclidean distance | Intercept (2014) | 31.257 | 0.716 | 43.643 | 82/95/123 | Individual identity, Nest identity |  |
|  | Degree | 0.000 | 0.000 | 0.000 |  |  |  |
|  | 2015 | -0.613 | 0.919 | -0.667 |  |  |  |
|  | 2018 | 0.448 | 1.293 | 0.347 |  |  |  |
| No. overlapping nests | Intercept (2014) | 3.846 | 0.055 | 70.743 | 82/95/123 | Individual identity, Nest identity |  |
|  | Strength | 0.007 | 0.029 | 0.25 |  |  |  |
|  | 2015 | -0.185 | 0.072 | -2.575 |  |  | |
|  | 2018 | -1.282 | 0.112 | -11.495 |  |  |  |
| Mean Euclidean distance | Intercept (2014) | 31.320 | 0.727 | 43.067 | 82/95/123 | Individual identity, Nest identity |  |
|  | Strength | 0.000 | 0.000 | 0.000 |  |  |  |
|  | 2015 | -0.420 | 0.929 | -0.452 |  |  |  |
|  | 2018 | 0.296 | 1.294 | 0.228 |  |  |  |

| **Table S6.** The relationship between the simple ratio index (*SRI*) between dyads in pre-breeding social networks and whether the nests of dyads temporally overlapped in incubation period and the Euclidean distance in metres between nests. Models included data from three years (2014, 2015 and 2018). Models of temporal overlap used a binomial error structure and Euclidean distances used Gaussian error structures with a log-link. *SRI* was scaled to have a mean of 0 and standard deviation of 1. Results are presented as treatment contrasts with respect to the intercept. *z*- and *t*-values are given for models of temporal overlap and Euclidean distance respectively. | | | | | | |
| --- | --- | --- | --- | --- | --- | --- |
| **Response** | **Fixed effects** | **Estimate** | **Standard Error** | ***z/t*-value** | **No. individuals/nests/**  **observations** | **Random effects** |
| Temporal overlap (0,1) | Intercept (2014, Grassland) | 1.460 | 0.309 | 4.727 | 61/59/1424 | Focal identity, Partner identity, Nest identity |
|  | Simple ratio index (*SRI*) | -0.051 | 0.061 | -0.835 |  |  |
|  | Habitat (Saltmarsh) | -0.049 | 0.307 | 0.161 |  |  |
|  | 2015 | -1.486 | 0.351 | -4.239 |  |  |
|  | 2018 | -1.265 | 0.613 | -2.464 |  |  |
| Euclidean distance | Intercept (2014, Grassland) | 6.606 | 0.0.73 | 90.250 | 61/59/1424 | Focal identity, Partner identity, Nest identity |
|  | Simple ratio index (*SRI*) | -0.01 | 0.01 | -0.952 |  |  |
|  | Habitat (Saltmarsh) | 0.294 | 0.076 | 3.869 |  |  |
|  | 2015 | -0.032 | 0.052 | -0.610 |  |  |
|  | 2018 | -0.385 | 0.069 | -5.605 |  |  |

| **Table S7.** Relationship between nest success, the number of temporally overlapping nests and the proximity to neighbouring nests. Model used binomial error structures and included data from 11 years. Results are shown for the full models including Julian date and habitat and are presented as treatment contrasts with respect to the intercept. | | | | | | | | |
| --- | --- | --- | --- | --- | --- | --- | --- | --- |
| **Response** | **Fixed effects** | **Estimate** | **Standard Error** | ***z*-value** | **No. of nests** | **Random effects** | |  |
| Nest success (0,1) | Intercept (Grassland) | -0.607 | 0.698 | -0.87 | 347 | Year | |  |
|  | No. overlapping nests | 0.026 | 0.009 | 2.887 |  |  | |  |
|  | Mean Euclidean distance | 0.001 | 0.001 | 1.3 |  |  | |  |
|  | Minimum Euclidean distance | -0.001 | 0.001 | -1.302 |  |  | |  |
|  | Habitat (Semi-desert) | -0.518 | 0.328 | -1.578 |  |  | |  |
|  | Habitat (Saltmarsh) | 0.092 | 0.371 | 0.247 |  |  | |  |
|  | Julian date | 0.047 | 0.155 | 0.305 |  |  | |  |
|  | Julian date^2 | -0.073 | 0.139 | -0.52 |  |  | |  |
| Nest success (0,1) | Intercept (Grassland) | 0.987 | 0.398 | 2.481 | 347 | Year | |  |
|  | No. overlapping nests <100m | 0.113 | 0.113 | 0.997 |  |  | |  |
|  | Habitat (Semi-desert) | -0.385 | 0.299 | -1.287 |  |  | |  |
|  | Habitat (Saltmarsh) | 0.207 | 0.329 | 0.631 |  |  | |  |
|  | Julian date | -0.098 | 0.142 | -0.687 |  |  | |  |
|  | Julian date^2 | -0.271 | 0.125 | -2.168 |  |  |  |  |

| **Table S8.** The relationship between the nesting success of individuals and the habitat of their previous nesting attempt and whether individuals disperse to a new habitat for their subsequent breeding attempt in the next year (Disperse) and the distance in metres between their consecutive nesting attempts. Models included data from 10 years. Model for dispersal used binomial error structures. Models for Distance between nests used Gaussian error structures with a log-link. Results are presented as treatment contrasts with respect to the intercept. *z*-values are given for model of dispersal and t-values are given for distance model. | | | | | | |  |  |
| --- | --- | --- | --- | --- | --- | --- | --- | --- |
| **Response** | **Fixed effects** | **Estimate** | **Standard Error** | ***z/t*-value** | **No. individuals/**  **observations** | **Random effects** | | |
| Disperse (0,1) | Intercept (Grassland) | 13.464 | 0.003 | 4595.013 | 68/86 | Individual identity, Year | | |
|  | Previous nest success | 17.400 | 0.003 | 5439.332 |  |  | | |
|  | Previous nesting habitat (Semi-desert) | -15.451 | 0.003 | -5029.756 |  |  | | |
|  | Previous nesting habitat (Saltmarsh) | 13.207 | 0.004 | 3530.149 |  |  | | |
| Distance between nests (m) | Intercept | 4.658 | 1.121 | 4.156 | 68/86 | Individual identity, Year | | |
|  | Previous nest success | -0.073 | 0.085 | -0.865 |  |  | | |
|  | Previous nesting habitat (Semi-desert) | -2.448 | 0.193 | -12.688 |  |  | | |
|  | Previous nesting habitat (Saltmarsh) | -0.562 | 0.035 | -15.949 |  |  | | |

| **Table S9.** The relationship between the distance between the nests of individuals across consecutive years and their previous nesting success, previous nesting habitat and the proportion of local neighbours within 100 metres that nested successfully during the previous breeding season. Data was included from across 10 years. Models used Gaussian error structures with a log-link. Results are presented as treatment contrasts with respect to the intercept. | | | | | | |
| --- | --- | --- | --- | --- | --- | --- |
| **Response** | **Parameter** | **Estimate** | **Standard Error** | ***t*-value** | **No. of nests** | **Random effects** |
| Distance between nests (m) | Intercept (Grassland) | 2.924 | 2.234 | 1.309 | 36 | - |
|  | Previous nest success | 0.641 | 0.698 | 0.918 |  |  |
|  | Previous nesting habitat (Semi-desert) | 1.467 | 0.617 | 2.376 |  |  |
|  | Previous nesting habitat (Saltmarsh) | -1.287 | 3.779 | -0.341 |  |  |
|  | Proportion neighbours successful | 1.466 | 2.073 | 0.707 |  |  |

**Supplementary references**

Carmona-Isunza, M. C., C. Küpper, M. A. Serrano-Meneses, and T. Székely. 2015. Courtship behavior differs between monogamous and polygamous plovers. Behav. Ecol. Sociobiol.

Franks, D. W., G. D. Ruxton, and R. James. 2010. Sampling animal association networks with the gambit of the group. Behav. Ecol. Sociobiol. 64:493–503.

Kosztolányi, A., S. Javed, C. Küpper, I. C. Cuthill, A. A. Shamsi, and T. Székely. 2009. Breeding ecology of Kentish Plover *Charadrius alexandrinus* in an extremely hot environment. Bird Study 56:244–252.

Krause, J., D. Lusseau, and R. James. 2009. Animal social networks: an introduction. Behav. Ecol. Sociobiol. 63:967–973.

Ramsar. 1971. Convention on Wetlands of International Importance Especially as Waterfowl Habitat. Ramsar, Iran.

RIS. 2013. Salinas of the English Port.

Robitaille, A. L., Q. M. R. Webber, and E. Vander Wal. 2018. Conducting social network analysis with animal telemetry data: applications and methods using spatsoc. bioRxiv, doi: https://doi.org/10.1101/447284.

Székely, T., A. Kosztolányi, and C. Küpper. 2008. Practical guide for investigating breeding ecology of Kentish plover *Charadrius alexandrinus*, v 3. Unpubl. Rep. Univ. Bath.
