# Supplementary material for: The impact of social structure on breeding strategies in an island bird
Source: Sci Rep. 2020 Aug 17;10:13872. doi: 10.1038/s41598-020-70595-w (PMC7431420; doi:10.1038/s41598-020-70595-w)
Supplement: Supplementary file 4 — Supplementary Data 2 [file 41598_2020_70595_MOESM4_ESM.pdf]

```r
# packages
library(dplyr)
library(igraph)

# Below are pre-breeding Kentish plover social networks from 2014,2015,2017 and 2018 in
igraph format.

#####
# social network 2014
#####
g2014 <- structure(list(114, FALSE, c(36, 2, 3, 93, 30, 25, 72, 100, 108,
44, 36, 83, 45, 61, 42, 85, 54, 63, 67, 103, 105, 38, 92, 62,
92, 42, 57, 84, 61, 78, 58, 95, 111, 80, 91, 84, 98, 113, 87,
91, 74, 74, 100, 108, 109, 91, 93, 107, 101, 112, 108, 108),
   c(0, 1, 1, 2, 5, 9, 11, 11, 11, 12, 15, 17, 19, 19, 21, 21,
   22, 23, 27, 29, 32, 34, 34, 35, 38, 40, 43, 43, 45, 48, 52,
   52, 53, 55, 55, 57, 58, 63, 66, 67, 69, 70, 72, 72, 77, 80,
   90, 95, 97, 99, 100, 102), c(1, 2, 5, 4, 0, 10, 21, 14, 25,
   9, 12, 16, 26, 30, 13, 28, 23, 17, 18, 6, 40, 41, 29, 33,
   11, 27, 35, 15, 38, 34, 39, 45, 22, 24, 3, 46, 31, 36, 7,
   42, 48, 19, 20, 47, 8, 43, 50, 51, 44, 32, 49, 37), c(0,
   1, 2, 3, 4, 5, 6, 7, 8, 9, 10, 11, 12, 13, 14, 15, 16, 17,
   18, 19, 20, 21, 22, 23, 24, 25, 26, 27, 28, 29, 30, 31, 32,
   33, 34, 35, 36, 37, 38, 39, 40, 41, 42, 43, 44, 45, 46, 47,
   48, 49, 50, 51), c(0, 0, 0, 1, 2, 2, 2, 2, 2, 2, 2, 2, 2,
   2, 2, 2, 2, 2, 2, 2, 2, 2, 2, 2, 2, 2, 3, 3, 3, 3, 3, 4,
   4, 4, 4, 4, 4, 6, 6, 7, 7, 7, 7, 9, 9, 10, 11, 11, 11, 11,
   11, 11, 11, 11, 11, 12, 12, 12, 13, 14, 14, 14, 16, 17, 18,
   18, 18, 18, 19, 19, 19, 19, 19, 20, 20, 22, 22, 22, 22, 23,
   23, 24, 24, 24, 25, 27, 28, 28, 29, 29, 29, 29, 32, 34, 36,
   36, 37, 37, 37, 38, 38, 40, 41, 41, 42, 42, 43, 43, 44, 48,
   49, 49, 50, 51, 52), c(0, 1, 3, 4, 4, 4, 5, 5, 5, 5, 6, 6,
   9, 10, 10, 10, 11, 11, 12, 12, 14, 14, 16, 17, 18, 18, 18,
   18, 19, 19, 20, 20, 20, 21, 21, 23, 24, 24, 24, 25, 25, 26,
   26, 26, 28, 28, 29, 29, 29, 30, 30, 30, 30, 32, 33, 33, 35,
   35, 36, 37, 37, 37, 37, 37, 38, 38, 38, 39, 40, 40, 41, 42,
   42, 44, 44, 44, 44, 44, 45, 45, 45, 46, 46, 46, 46, 46, 46,
   46, 46, 46, 46, 47, 47, 47, 47, 47, 48, 48, 49, 49, 50, 51,
   51, 52, 52, 52, 52, 52, 52, 52, 52, 52, 52, 52, 52), list(
      c(1, 0, 1), structure(list(), .Names = character(0)),
      list(name = c("CA1052", "CA1058", "CA1059", "CA1062",
      "CA1067", "CA1089", "CA1096", "CA1104", "CA1111", "CA1119",
      "CA1126", "CA1137", "CA1180", "CA1619", "CA1624", "CA1630",
      "CA1635", "CA1639", "CA1641", "CA1644", "CA1647", "CA1663",
      "CA1676", "CA1693", "CA1699", "CA2629", "CA2686", "CA2691",
```

```
"CA2694", "CA2712", "CA2714", "CA2721", "CA2727", "CA2745",
"CA2753", "CA2754", "CA2756", "CA2757", "CA2758", "CA2767",
"CA2770", "CA2782", "CA3003", "CA3010", "CA3016", "CA3017",
"CA3027", "CA3036", "CA3049", "CA3056", "CA3058", "CA3066",
"CA3071", "CA3080", "CA3082", "CA3083", "CA3084", "CA3085",
"CA3095", "CA3121", "CA3130", "CA3140", "CA3141", "CA3145",
"CA3146", "CA3154", "CA3174", "CA3183", "CA3405", "CA3424",
"CA3425", "CA3427", "CA3428", "CA3435", "CA3440", "CA3447",
"CA3460", "CA3464", "CA3605", "CA3606", "CA3612", "CA3616",
"CA3617", "CA3621", "CA3655", "CA3666", "CA3753", "CA3754",
"CA3757", "CA3760", "CA3764", "CA3765", "CA3770", "CA3774",
"CA3787", "CA3789", "CA3793", "CA3807", "CA3809", "CA3814",
"CA3826", "CA3838", "CA3849", "CA3861", "CA3866", "CA3869",
"CA4008", "CA4036", "CA4078", "CA4083", "CA4088", "CA4095",
"CA4099", "CA4384"), `TRUE` = c("CA1052", "CA1058", "CA1059",
"CA1062", "CA1067", "CA1089", "CA1096", "CA1104", "CA1111",
"CA1119", "CA1126", "CA1137", "CA1180", "CA1619", "CA1624",
"CA1630", "CA1635", "CA1639", "CA1641", "CA1644", "CA1647",
"CA1663", "CA1676", "CA1693", "CA1699", "CA2629", "CA2686",
"CA2691", "CA2694", "CA2712", "CA2714", "CA2721", "CA2727",
"CA2745", "CA2753", "CA2754", "CA2756", "CA2757", "CA2758",
"CA2767", "CA2770", "CA2782", "CA3003", "CA3010", "CA3016",
"CA3017", "CA3027", "CA3036", "CA3049", "CA3056", "CA3058",
"CA3066", "CA3071", "CA3080", "CA3082", "CA3083", "CA3084",
"CA3085", "CA3095", "CA3121", "CA3130", "CA3140", "CA3141",
"CA3145", "CA3146", "CA3154", "CA3174", "CA3183", "CA3405",
"CA3424", "CA3425", "CA3427", "CA3428", "CA3435", "CA3440",
"CA3447", "CA3460", "CA3464", "CA3605", "CA3606", "CA3612",
"CA3616", "CA3617", "CA3621", "CA3655", "CA3666", "CA3753",
"CA3754", "CA3757", "CA3760", "CA3764", "CA3765", "CA3770",
"CA3774", "CA3787", "CA3789", "CA3793", "CA3807", "CA3809",
"CA3814", "CA3826", "CA3838", "CA3849", "CA3861", "CA3866",
"CA3869", "CA4008", "CA4036", "CA4078", "CA4083", "CA4088",
"CA4095", "CA4099", "CA4384"), sex = c(2L, 1L, 2L, 1L,
1L, 1L, 2L, 1L, 1L, 2L, 2L, 1L, 2L, 2L, 2L, 2L, 2L, 2L,
2L, 1L, 2L, 2L, 2L, 1L, 2L, 1L, 2L, 1L, 2L, 1L, 2L, 1L,
1L, 2L, 1L, 2L, 1L, 1L, 2L, 2L, 1L, 2L, 1L, 1L, 1L, 2L,
2L, 2L, 1L, 1L, 2L, 2L, 1L, 1L, 2L, 1L, 2L, 1L, 1L, 1L,
2L, 2L, 1L, 2L, 1L, 1L, 2L, 2L, 1L, 1L, 1L, 1L, 1L, 1L,
2L, 1L, 2L, 1L, 2L, 1L, 2L, 2L, 2L, 1L, 1L, 2L, 2L, 1L,
2L, 2L, 1L, 1L, 1L, 2L, 1L, 2L, 1L, 1L, 2L, 1L, 2L, 2L,
1L, 2L, 2L, 2L, 2L, 1L, 2L, 2L, 2L, 2L, 2L, 2L), cols = c("white",
"white", "white", "white", "white", "white", "white",
"white", "white", "white", "white", "white", "white",
"white", "white", "white", "white", "white", "white",
"white", "white", "white", "white", "white", "white",
"white", "white", "white", "white", "white", "white",
```

```
    "white", "white", "white", "white", "white", "white",
    "white", "white", "white", "white", "white", "white",
    "white", "white", "white", "white", "white", "white",
    "white", "white", "white", "white", "white", "white",
    "white", "white", "white", "white", "white", "white",
    "white", "white", "white", "white", "white", "white",
    "white", "white", "white", "white", "white", "white",
    "white", "white", "white", "white", "white", "white",
    "white", "white", "white", "white", "white", "white",
    "white", "white", "white", "white", "white", "white",
    "white", "white", "white", "white", "white", "white",
    "white", "white", "white", "white", "white", "white",
    "white", "white", "white", "white", "white", "white",
    "white", "white", "white", "white", "white")), list(weight = c(0.5,
    0.25, 0.333333333333333, 0.166666666666667, 0.8, 0.5,
    1, 0.5, 0.5, 1, 0.333333333333333, 0.333333333333333,
    1, 1, 0.166666666666667, 0.142857142857143, 0.333333333333333,
    0.75, 0.25, 1, 0.5, 1, 1, 0.25, 1, 0.333333333333333,
    0.5, 0.5, 1, 0.5, 0.0909090909090909, 0.1, 1, 0.5, 0.5,
    1, 0.125, 0.25, 0.5, 0.25, 0.333333333333333, 0.5, 0.5,
    0.5, 1, 0.333333333333333, 0.5, 0.25, 0.666666666666667,
    0.5, 0.333333333333333, 0.5)))), class = "igraph")

#####
# social network 2015
#####
g2015 <- structure(list(126, FALSE, c(115, 92, 55, 76, 89, 39, 41, 47,
66, 105, 38, 41, 119, 106, 13, 83, 87, 108, 112, 83, 87, 108,
112, 59, 65, 83, 88, 66, 83, 99, 70, 121, 121, 29, 30, 68, 72,
102, 68, 102, 72, 73, 44, 110, 115, 68, 84, 88, 108, 40, 41,
110, 115, 119, 41, 78, 83, 110, 115, 61, 72, 119, 99, 124, 44,
50, 53, 106, 111, 53, 110, 111, 115, 69, 115, 53, 106, 110, 110,
106, 112, 76, 90, 91, 57, 61, 117, 118, 65, 83, 88, 72, 118,
104, 86, 87, 83, 88, 97, 101, 102, 94, 121, 89, 83, 87, 88, 117,
87, 88, 108, 112, 88, 108, 100, 101, 103, 107, 87, 97, 97, 108,
112, 108, 91, 124, 101, 103, 107, 103, 107, 107, 112, 110, 115,
119, 125, 124), c(0, 3, 4, 4, 4, 5, 5, 6, 6, 8, 9, 9, 9, 10,
12, 12, 12, 12, 12, 13, 13, 13, 13, 16, 16, 16, 16, 18, 19, 20,
24, 24, 26, 27, 27, 27, 27, 27, 29, 29, 30, 31, 35, 35, 35, 36,
36, 36, 36, 38, 38, 38, 38, 38, 39, 39, 39, 40, 40, 41, 41, 41,
42, 42, 43, 43, 43, 43, 43, 44, 44, 44, 44, 46, 49, 50, 50, 50,
52, 53, 54, 55, 55, 55, 56, 56, 56, 56, 59, 59, 59, 61, 61, 62,
64, 64, 65, 65, 67, 68, 68, 69, 70, 76, 78, 79, 79, 79, 83, 83,
83, 83, 84, 84, 85, 85, 85, 85, 86, 86, 87, 87, 87, 88, 90, 99,
100, 100, 100, 101, 101, 103, 108, 109, 110, 113, 116, 123),
  c(14, 33, 34, 10, 5, 49, 6, 11, 50, 54, 42, 64, 7, 65, 66,
  69, 75, 2, 84, 23, 59, 85, 24, 88, 8, 27, 35, 38, 45, 73,
```

```
30, 36, 40, 60, 91, 41, 3, 81, 55, 15, 19, 25, 28, 56, 89,
96, 104, 46, 94, 16, 20, 95, 105, 108, 118, 26, 47, 90, 97,
106, 109, 112, 4, 103, 82, 83, 124, 1, 101, 98, 119, 120,
29, 62, 114, 99, 115, 126, 37, 39, 100, 116, 127, 129, 93,
9, 13, 67, 76, 79, 117, 128, 130, 131, 17, 21, 48, 110, 113,
121, 123, 43, 51, 57, 70, 77, 78, 133, 68, 71, 18, 22, 80,
111, 122, 132, 0, 44, 52, 58, 72, 74, 134, 86, 107, 87, 92,
12, 53, 61, 135, 31, 32, 102, 63, 125, 137, 136), c(0, 1,
2, 3, 4, 5, 6, 7, 8, 9, 10, 11, 12, 13, 14, 15, 16, 17, 18,
19, 20, 21, 22, 23, 24, 25, 26, 27, 28, 29, 30, 31, 32, 33,
34, 35, 36, 37, 38, 39, 40, 41, 42, 43, 44, 45, 46, 47, 48,
49, 50, 51, 52, 53, 54, 55, 56, 57, 58, 59, 60, 61, 62, 63,
64, 65, 66, 67, 68, 69, 70, 71, 72, 73, 74, 75, 76, 77, 78,
79, 80, 81, 82, 83, 84, 85, 86, 87, 88, 89, 90, 91, 92, 93,
94, 95, 96, 97, 98, 99, 100, 101, 102, 103, 104, 105, 106,
107, 108, 109, 110, 111, 112, 113, 114, 115, 116, 117, 118,
119, 120, 121, 122, 123, 124, 125, 126, 127, 128, 129, 130,
131, 132, 133, 134, 135, 136, 137), c(0, 0, 0, 0, 0, 0, 0,
0, 0, 0, 0, 0, 0, 0, 1, 1, 1, 1, 1, 1, 1, 1, 1, 1, 1,
1, 1, 1, 1, 2, 3, 3, 3, 3, 3, 3, 3, 3, 4, 5, 6, 10, 10, 10,
12, 12, 12, 13, 13, 13, 14, 14, 14, 17, 17, 18, 18, 19, 19,
20, 20, 22, 22, 22, 22, 24, 26, 26, 29, 30, 31, 31, 35, 36,
36, 36, 38, 38, 39, 39, 39, 39, 39, 47, 48, 48, 49, 55, 62,
64, 65, 67, 68, 68, 69, 69, 69, 72, 72, 74, 75, 78, 81, 84,
85, 86, 90, 94, 101, 101, 108, 110, 116, 116, 116, 123, 123,
125, 127, 131, 131, 134, 134, 134, 137, 138), c(0, 1, 1,
1, 2, 5, 7, 9, 9, 10, 13, 14, 14, 19, 23, 23, 23, 27, 27,
28, 29, 30, 30, 30, 30, 32, 32, 33, 38, 38, 40, 41, 42, 42,
42, 42, 45, 49, 49, 54, 57, 59, 62, 64, 69, 73, 73, 74, 74,
74, 75, 78, 78, 79, 80, 81, 84, 88, 88, 88, 91, 91, 93, 94,
94, 96, 98, 98, 99, 101, 102, 103, 103, 103, 103, 103, 103,
104, 104, 105, 108, 108, 108, 108, 112, 114, 118, 120, 123,
124, 124, 125, 125, 125, 125, 125, 125, 125, 125, 125, 126,
129, 131, 131, 132, 132, 132, 132, 132, 133, 134, 135, 135,
135, 136, 136, 136, 137, 137, 137, 137, 137, 137, 137, 138,
138, 138), list(c(1, 0, 1), structure(list(), .Names = character(0)),
    list(name = c("CA1018", "CA1044", "CA1047", "CA1052",
    "CA1067", "CA1087", "CA1092", "CA1093", "CA1126", "CA1169",
    "CA1626", "CA1630", "CA1639", "CA1663", "CA1670", "CA1674",
    "CA1676", "CA1687", "CA1693", "CA1694", "CA1697", "CA2619",
    "CA2626", "CA2653", "CA2666", "CA2672", "CA2683", "CA2686",
    "CA2688", "CA2691", "CA2711", "CA2714", "CA2721", "CA2727",
    "CA2741", "CA2744", "CA2745", "CA2746", "CA2753", "CA2757",
    "CA2758", "CA2762", "CA2764", "CA2780", "CA2782", "CA2783",
    "CA2795", "CA3010", "CA3015", "CA3017", "CA3027", "CA3036",
    "CA3054", "CA3058", "CA3065", "CA3071", "CA3075", "CA3076",
    "CA3077", "CA3082", "CA3084", "CA3085", "CA3095", "CA3103",
```

```
"CA3121", "CA3141", "CA3145", "CA3174", "CA3183", "CA3194",
"CA3405", "CA3423", "CA3427", "CA3431", "CA3440", "CA3448",
"CA3460", "CA3605", "CA3606", "CA3607", "CA3612", "CA3614",
"CA3617", "CA3621", "CA3622", "CA3623", "CA3655", "CA3658",
"CA3663", "CA3665", "CA3666", "CA3668", "CA3680", "CA3695",
"CA3696", "CA3697", "CA3753", "CA3754", "CA3755", "CA3757",
"CA3760", "CA3764", "CA3765", "CA3774", "CA3787", "CA3789",
"CA3792", "CA3793", "CA3807", "CA3809", "CA3814", "CA3826",
"CA3838", "CA3850", "CA3861", "CA3869", "CA4008", "CA4022",
"CA4077", "CA4088", "CA4095", "CA4099", "CA4100", "CA4166",
"CA4167", "CA4317"), `TRUE` = c("CA1018", "CA1044", "CA1047",
"CA1052", "CA1067", "CA1087", "CA1092", "CA1093", "CA1126",
"CA1169", "CA1626", "CA1630", "CA1639", "CA1663", "CA1670",
"CA1674", "CA1676", "CA1687", "CA1693", "CA1694", "CA1697",
"CA2619", "CA2626", "CA2653", "CA2666", "CA2672", "CA2683",
"CA2686", "CA2688", "CA2691", "CA2711", "CA2714", "CA2721",
"CA2727", "CA2741", "CA2744", "CA2745", "CA2746", "CA2753",
"CA2757", "CA2758", "CA2762", "CA2764", "CA2780", "CA2782",
"CA2783", "CA2795", "CA3010", "CA3015", "CA3017", "CA3027",
"CA3036", "CA3054", "CA3058", "CA3065", "CA3071", "CA3075",
"CA3076", "CA3077", "CA3082", "CA3084", "CA3085", "CA3095",
"CA3103", "CA3121", "CA3141", "CA3145", "CA3174", "CA3183",
"CA3194", "CA3405", "CA3423", "CA3427", "CA3431", "CA3440",
"CA3448", "CA3460", "CA3605", "CA3606", "CA3607", "CA3612",
"CA3614", "CA3617", "CA3621", "CA3622", "CA3623", "CA3655",
"CA3658", "CA3663", "CA3665", "CA3666", "CA3668", "CA3680",
"CA3695", "CA3696", "CA3697", "CA3753", "CA3754", "CA3755",
"CA3757", "CA3760", "CA3764", "CA3765", "CA3774", "CA3787",
"CA3789", "CA3792", "CA3793", "CA3807", "CA3809", "CA3814",
"CA3826", "CA3838", "CA3850", "CA3861", "CA3869", "CA4008",
"CA4022", "CA4077", "CA4088", "CA4095", "CA4099", "CA4100",
"CA4166", "CA4167", "CA4317"), sex = c(1L, 1L, 2L, 2L,
1L, 2L, 1L, 2L, 2L, 2L, 1L, 2L, 2L, 2L, 1L, 1L, 2L, 2L,
1L, 2L, 1L, 2L, 2L, 2L, 2L, 2L, 1L, 2L, 1L, 1L, 2L, 2L,
1L, 1L, 2L, 1L, 2L, 1L, 1L, 1L, 2L, 1L, 1L, 1L, 2L, 2L,
2L, 1L, 2L, 2L, 2L, 2L, 1L, 2L, 2L, 1L, 2L, 1L, 2L, 2L,
2L, 1L, 1L, 1L, 1L, 1L, 2L, 2L, 2L, 1L, 1L, 2L, 1L, 1L,
2L, 1L, 2L, 2L, 1L, 1L, 2L, 1L, 2L, 1L, 2L, 1L, 1L, 1L,
1L, 1L, 2L, 1L, 1L, 1L, 2L, 1L, 2L, 1L, 2L, 2L, 2L, 1L,
1L, 2L, 1L, 2L, 2L, 1L, 1L, 2L, 1L, 2L, 2L, 1L, 2L, 2L,
2L, 1L, 2L, 2L, 2L, 2L, 1L, 2L, 1L, 2L), cols = c("white",
"white", "white", "white", "white", "white", "white",
"white", "white", "white", "white", "white", "white",
"white", "white", "white", "white", "white", "white",
"white", "white", "white", "white", "white", "white",
"white", "white", "white", "white", "white", "white",
"white", "white", "white", "white", "white", "white",
```

```
    "white", "white", "white", "white", "white", "white",
    "white", "white", "white", "white", "white", "white",
    "white", "white", "white", "white", "white", "white",
    "white", "white", "white", "white", "white", "white",
    "white", "white", "white", "white", "white", "white",
    "white", "white", "white", "white", "white", "white",
    "white", "white", "white", "white", "white", "white",
    "white", "white", "white", "white", "white", "white",
    "white", "white", "white", "white", "white", "white",
    "white", "white", "white", "white", "white", "white",
    "white", "white", "white", "white", "white", "white",
    "white", "white", "white", "white", "white", "white",
    "white", "white", "white", "white", "white", "white",
    "white", "white", "white", "white", "white", "white",
    "white", "white", "white", "white", "white")), list(weight = c(0.2,
    1, 0.166666666666667, 0.6, 0.2, 0.5, 0.2, 0.5, 0.2, 1,
    0.25, 0.2, 0.333333333333333, 0.5, 0.333333333333333,
    0.166666666666667, 0.166666666666667, 0.25, 0.333333333333333,
    0.125, 0.125, 0.166666666666667, 0.2, 0.25, 0.5, 0.125,
    0.2, 0.4, 0.166666666666667, 0.333333333333333, 1, 0.333333333333333,
    0.333333333333333, 0.5, 0.333333333333333, 0.2, 0.333333333333333,
    0.333333333333333, 0.25, 0.5, 0.333333333333333, 0.333333333333333,
    0.2, 0.2, 0.2, 0.125, 0.2, 0.142857142857143, 0.125,
    0.25, 0.125, 0.125, 0.125, 0.166666666666667, 0.166666666666667,
    0.5, 0.142857142857143, 0.2, 0.2, 0.166666666666667,
    0.166666666666667, 0.142857142857143, 0.142857142857143,
    0.142857142857143, 0.166666666666667, 0.2, 0.25, 0.333333333333333,
    0.5, 0.142857142857143, 0.111111111111111, 0.2, 0.25,
    0.5, 0.2, 0.166666666666667, 0.2, 0.125, 0.2, 0.25, 0.333333333333333,
    0.25, 0.5, 0.5, 0.166666666666667, 0.166666666666667,
    0.166666666666667, 0.2, 0.25, 0.142857142857143, 0.25,
    0.333333333333333, 0.5, 0.333333333333333, 0.333333333333333,
    0.166666666666667, 0.125, 0.2, 0.25, 0.2, 0.5, 0.5, 0.333333333333333,
    0.333333333333333, 0.166666666666667, 0.125, 0.2, 0.25,
    0.0909090909090909, 0.125, 0.25, 0.125, 0.333333333333333,
    0.25, 0.25, 0.333333333333333, 0.25, 0.2, 0.5, 0.166666666666667,
    0.111111111111111, 0.111111111111111, 0.125, 0.166666666666667,
    1, 0.2, 0.25, 0.2, 0.4, 0.25, 0.2, 0.4, 0.166666666666667,
    0.125, 0.25, 0.333333333333333, 1, 0.333333333333333)))), class = "igraph")


#####
# social network 2017
#####
g2017 <- structure(list(152, FALSE, c(20, 136, 84, 96, 97, 89, 102, 124,
```

28, 93, 119, 121, 149, 23, 81, 107, 108, 12, 124, 121, 149, 61,
16, 68, 112, 92, 136, 40, 115, 81, 107, 108, 95, 102, 149, 149,
74, 109, 128, 117, 125, 127, 142, 84, 75, 49, 75, 115, 149, 117,
133, 72, 66, 143, 109, 144, 148, 110, 117, 144, 70, 88, 132,
90, 129, 122, 131, 109, 128, 139, 110, 133, 148, 109, 113, 108,
107, 108, 98, 147, 121, 121, 149, 101, 117, 149, 102, 97, 117,
125, 126, 127, 133, 106, 113, 106, 108, 113, 128, 117, 144, 114,
125, 126, 127, 133, 142, 144, 121, 129, 149, 141, 127, 142, 127,
133, 133, 142, 134, 142, 148, 136, 149, 148), c(0, 0, 2, 2, 2,
3, 3, 5, 6, 6, 6, 6, 6, 7, 7, 7, 7, 8, 9, 11, 11, 12, 13, 15,
16, 20, 20, 21, 21, 23, 23, 23, 25, 25, 26, 28, 29, 29, 29, 30,
30, 30, 30, 34, 35, 37, 40, 40, 40, 41, 41, 43, 44, 46, 48, 49,
49, 51, 51, 51, 52, 53, 54, 55, 58, 68, 72, 74, 74, 74, 75, 77,
77, 78, 78, 79, 81, 81, 82, 86, 88, 91, 91, 93, 93, 93, 95, 96,
100, 100, 100, 100, 100, 102, 103, 105, 107, 109, 109, 110, 110,
112, 117, 117, 117, 117, 117, 117, 119, 120, 121, 123, 125, 125,
126, 126, 127, 127, 130, 133, 133, 135, 138, 144), c(17, 22,
0, 13, 8, 27, 45, 21, 52, 23, 60, 51, 36, 44, 46, 14, 29, 2,
43, 61, 5, 63, 25, 9, 32, 3, 4, 87, 78, 83, 6, 33, 86, 93, 95,
15, 30, 76, 16, 31, 75, 77, 96, 37, 54, 67, 73, 57, 70, 24, 74,
94, 97, 101, 28, 47, 39, 49, 58, 84, 88, 99, 10, 11, 19, 80,
81, 108, 65, 7, 18, 40, 89, 102, 90, 103, 41, 91, 104, 112, 114,
38, 68, 98, 64, 109, 66, 62, 50, 71, 92, 105, 115, 116, 118,
1, 26, 121, 69, 111, 42, 106, 113, 117, 119, 53, 55, 59, 100,
107, 79, 56, 72, 120, 123, 12, 20, 34, 35, 48, 82, 85, 110, 122
), c(0, 1, 2, 3, 4, 5, 6, 7, 8, 9, 10, 11, 12, 13, 14, 15, 16,
17, 18, 19, 20, 21, 22, 23, 24, 25, 26, 27, 28, 29, 30, 31, 32,
33, 34, 35, 36, 37, 38, 39, 40, 41, 42, 43, 44, 45, 46, 47, 48,
49, 50, 51, 52, 53, 54, 55, 56, 57, 58, 59, 60, 61, 62, 63, 64,
65, 66, 67, 68, 69, 70, 71, 72, 73, 74, 75, 76, 77, 78, 79, 80,
81, 82, 83, 84, 85, 86, 87, 88, 89, 90, 91, 92, 93, 94, 95, 96,
97, 98, 99, 100, 101, 102, 103, 104, 105, 106, 107, 108, 109,
110, 111, 112, 113, 114, 115, 116, 117, 118, 119, 120, 121, 122,
123), c(0, 0, 0, 0, 0, 0, 0, 0, 0, 0, 0, 0, 0, 1, 1, 1, 1, 2,
2, 2, 2, 3, 3, 3, 4, 4, 4, 4, 4, 5, 5, 5, 5, 5, 5, 5, 5, 5, 5,
5, 5, 6, 6, 6, 6, 6, 6, 6, 6, 7, 7, 7, 7, 7, 7, 7, 7, 7, 7,
7, 7, 8, 8, 8, 8, 8, 9, 9, 10, 10, 11, 11, 12, 12, 13, 15, 15,
15, 15, 15, 15, 17, 17, 17, 19, 19, 19, 19, 20, 21, 22, 22, 23,
24, 24, 25, 26, 28, 29, 29, 29, 30, 33, 33, 33, 33, 35, 38, 43,
47, 49, 49, 50, 53, 54, 56, 56, 62, 62, 63, 63, 68, 69, 69, 71,
74, 76, 81, 84, 86, 86, 87, 88, 94, 95, 95, 98, 98, 98, 99, 99,
100, 105, 106, 110, 110, 110, 111, 115, 124, 124, 124), c(0,
2, 2, 5, 7, 7, 8, 13, 17, 18, 19, 19, 21, 22, 23, 23, 24, 25,
25, 25, 25, 27, 29, 29, 32, 32, 34, 35, 35, 36, 39, 43, 43, 43,
43, 44, 45, 45, 46, 46, 46, 49, 51, 51, 52, 53, 53, 54, 54, 55,
57, 57, 60, 61, 62, 63, 64, 64, 64, 65, 65, 65, 65, 65, 65, 65,
65, 65, 65, 66, 66, 66, 66, 67, 67, 70, 71, 71, 73, 75, 76, 76,

```
78, 79, 79, 79, 79, 80, 80, 81, 81, 81, 83, 83, 86, 86, 87, 88,
88, 88, 88, 93, 93, 94, 95, 95, 96, 96, 97, 97, 99, 101, 101,
102, 102, 102, 102, 102, 108, 108, 109, 110, 111, 111, 112, 112,
114, 116, 118, 118, 118, 119, 119, 119, 121, 121, 122, 122, 122,
123, 123, 123, 123, 123, 123, 124, 124, 124, 124, 124, 124, 124,
124), list(c(1, 0, 1), structure(list(), .Names = character(0)),
    list(name = c("CA1047", "CA1059", "CA1067", "CA1080", "CA1089",
    "CA1092", "CA1093", "CA1119", "CA1126", "CA1177", "CA1635",
    "CA1637", "CA1641", "CA1644", "CA1651", "CA1662", "CA1663",
    "CA1676", "CA1683", "CA1687", "CA1694", "CA1699", "CA2628",
    "CA2629", "CA2651", "CA2672", "CA2686", "CA2691", "CA2703",
    "CA2706", "CA2711", "CA2714", "CA2715", "CA2721", "CA2725",
    "CA2727", "CA2738", "CA2744", "CA2745", "CA2746", "CA2753",
    "CA2754", "CA2757", "CA2762", "CA2764", "CA2767", "CA2774",
    "CA2776", "CA2780", "CA2782", "CA2783", "CA3003", "CA3015",
    "CA3016", "CA3017", "CA3026", "CA3027", "CA3036", "CA3046",
    "CA3058", "CA3060", "CA3062", "CA3065", "CA3066", "CA3071",
    "CA3075", "CA3082", "CA3083", "CA3084", "CA3085", "CA3103",
    "CA3121", "CA3130", "CA3139", "CA3141", "CA3145", "CA3154",
    "CA3183", "CA3194", "CA3195", "CA3417", "CA3418", "CA3424",
    "CA3425", "CA3460", "CA3464", "CA3604", "CA3606", "CA3607",
    "CA3616", "CA3621", "CA3622", "CA3638", "CA3655", "CA3662",
    "CA3665", "CA3666", "CA3668", "CA3696", "CA3697", "CA3708",
    "CA3751", "CA3753", "CA3754", "CA3758", "CA3764", "CA3774",
    "CA3787", "CA3789", "CA3792", "CA3793", "CA3801", "CA3807",
    "CA3826", "CA3838", "CA3849", "CA3868", "CA3869", "CA3879",
    "CA3901", "CA3903", "CA3934", "CA3949", "CA3956", "CA3964",
    "CA3974", "CA4006", "CA4008", "CA4010", "CA4022", "CA4025",
    "CA4039", "CA4043", "CA4050", "CA4057", "CA4058", "CA4059",
    "CA4077", "CA4088", "CA4095", "CA4101", "CA4102", "CA4136",
    "CA4142", "CA4157", "CA4166", "CA4175", "CA4343", "CA4382",
    "CA4383", "CA4389", "CA4595"), `TRUE` = c("CA1047", "CA1059",
    "CA1067", "CA1080", "CA1089", "CA1092", "CA1093", "CA1119",
    "CA1126", "CA1177", "CA1635", "CA1637", "CA1641", "CA1644",
    "CA1651", "CA1662", "CA1663", "CA1676", "CA1683", "CA1687",
    "CA1694", "CA1699", "CA2628", "CA2629", "CA2651", "CA2672",
    "CA2686", "CA2691", "CA2703", "CA2706", "CA2711", "CA2714",
    "CA2715", "CA2721", "CA2725", "CA2727", "CA2738", "CA2744",
    "CA2745", "CA2746", "CA2753", "CA2754", "CA2757", "CA2762",
    "CA2764", "CA2767", "CA2774", "CA2776", "CA2780", "CA2782",
    "CA2783", "CA3003", "CA3015", "CA3016", "CA3017", "CA3026",
    "CA3027", "CA3036", "CA3046", "CA3058", "CA3060", "CA3062",
    "CA3065", "CA3066", "CA3071", "CA3075", "CA3082", "CA3083",
    "CA3084", "CA3085", "CA3103", "CA3121", "CA3130", "CA3139",
    "CA3141", "CA3145", "CA3154", "CA3183", "CA3194", "CA3195",
    "CA3417", "CA3418", "CA3424", "CA3425", "CA3460", "CA3464",
    "CA3604", "CA3606", "CA3607", "CA3616", "CA3621", "CA3622",
```

```
"CA3638", "CA3655", "CA3662", "CA3665", "CA3666", "CA3668",
"CA3696", "CA3697", "CA3708", "CA3751", "CA3753", "CA3754",
"CA3758", "CA3764", "CA3774", "CA3787", "CA3789", "CA3792",
"CA3793", "CA3801", "CA3807", "CA3826", "CA3838", "CA3849",
"CA3868", "CA3869", "CA3879", "CA3901", "CA3903", "CA3934",
"CA3949", "CA3956", "CA3964", "CA3974", "CA4006", "CA4008",
"CA4010", "CA4022", "CA4025", "CA4039", "CA4043", "CA4050",
"CA4057", "CA4058", "CA4059", "CA4077", "CA4088", "CA4095",
"CA4101", "CA4102", "CA4136", "CA4142", "CA4157", "CA4166",
"CA4175", "CA4343", "CA4382", "CA4383", "CA4389", "CA4595"
), sex = c(2L, 2L, 1L, 1L, 1L, 1L, 2L, 2L, 2L, 1L, 2L, 1L,
2L, 1L, 1L, 1L, 2L, 2L, 1L, 2L, 2L, 2L, 1L, 1L, 2L, 2L, 2L,
1L, 1L, 2L, 2L, 2L, 1L, 1L, 1L, 1L, 1L, 1L, 2L, 1L, 1L, 2L,
1L, 1L, 1L, 2L, 1L, 2L, 1L, 2L, 2L, 1L, 2L, 1L, 2L, 2L, 2L,
2L, 2L, 2L, 1L, 1L, 2L, 2L, 1L, 2L, 2L, 1L, 2L, 1L, 1L, 1L,
2L, 1L, 1L, 2L, 1L, 2L, 1L, 2L, 1L, 1L, 1L, 1L, 2L, 1L, 2L,
1L, 1L, 2L, 1L, 2L, 2L, 1L, 2L, 1L, 2L, 1L, 2L, 1L, 1L, 2L,
2L, 1L, 2L, 1L, 2L, 1L, 2L, 2L, 1L, 1L, 1L, 2L, 2L, 1L, 1L,
2L, 2L, 1L, 1L, 2L, 2L, 2L, 2L, 2L, 1L, 2L, 2L, 1L, 2L, 2L,
2L, 2L, 1L, 2L, 1L, 2L, 2L, 2L, 2L, 1L, 2L, 2L, 1L, 2L, 2L,
1L, 1L, 2L, 2L, 1L), cols = c("white", "white", "white",
"white", "white", "white", "white", "white", "white", "white",
"white", "white", "white", "white", "white", "white", "white",
"white", "white", "white", "white", "white", "white", "white",
"white", "white", "white", "white", "white", "white", "white",
"white", "white", "white", "white", "white", "white", "white",
"white", "white", "white", "white", "white", "white", "white",
"white", "white", "white", "white", "white", "white", "white",
"white", "white", "white", "white", "white", "white", "white",
"white", "white", "white", "white", "white", "white", "white",
"white", "white", "white", "white", "white", "white", "white",
"white", "white", "white", "white", "white", "white", "white",
"white", "white", "white", "white", "white", "white", "white",
"white", "white", "white", "white", "white", "white", "white",
"white", "white", "white", "white", "white", "white", "white",
"white", "white", "white", "white", "white", "white", "white",
"white", "white", "white", "white", "white", "white", "white",
"white", "white", "white", "white", "white", "white", "white",
"white", "white", "white", "white", "white", "white", "white",
"white", "white", "white", "white", "white", "white", "white",
"white", "white", "white", "white", "white", "white", "white",
"white", "white", "white", "white", "white", "white", "white",
"white", "white", "white", "white", "white", "white", "white",
"white", "white", "white", "white", "white", "white", "white",
"white", "white")), list(weight = c(0.142857142857143, 0.285714285714286,
0.0769230769230769, 0.166666666666667, 0.166666666666667,
0.142857142857143, 0.111111111111111, 0.142857142857143,
0.2, 0.111111111111111, 0.2, 0.125, 0.0666666666666667, 1,
1, 0.25, 0.2, 0.5, 0.285714285714286, 0.25, 0.0909090909090909,
```

```
    0.5, 0.166666666666667, 0.0833333333333333, 0.1, 0.25, 0.125,
    0.125, 0.25, 1, 0.25, 0.2, 0.25, 0.166666666666667, 0.0769230769230769,
    0.0909090909090909, 0.5, 0.166666666666667, 1, 0.125, 0.2,
    0.2, 0.5, 0.125, 0.125, 0.125, 0.0769230769230769, 0.142857142857143,
    0.0625, 0.125, 0.25, 0.0909090909090909, 0.5, 1, 0.142857142857143,
    0.4, 0.0909090909090909, 0.2, 0.125, 0.166666666666667, 0.5,
    0.5, 1, 0.25, 0.125, 0.0588235294117647, 0.166666666666667,
    0.142857142857143, 0.5, 0.333333333333333, 0.0833333333333333,
    0.2, 0.2, 0.166666666666667, 0.2, 0.166666666666667, 0.25,
    0.2, 0.5, 0.5, 0.2, 0.25, 0.0909090909090909, 0.2, 0.0833333333333333,
    0.0666666666666667, 0.428571428571429, 1, 0.2, 0.125, 0.25,
    0.125, 0.142857142857143, 0.111111111111111, 0.181818181818182,
    0.4, 0.285714285714286, 0.1, 0.166666666666667, 0.0833333333333333,
    0.1, 0.5, 0.181818181818182, 0.125, 0.181818181818182, 0.2,
    0.111111111111111, 0.0769230769230769, 0.25, 0.125, 0.153846153846154,
    1, 0.25, 0.166666666666667, 0.2, 0.25, 0.125, 0.166666666666667,
    1, 0.2, 0.142857142857143, 0.142857142857143, 0.0769230769230769,
    0.111111111111111)))), class = "igraph")


#####
# social network 2018
#####
g2018 <- structure(list(156, FALSE, c(16, 9, 104, 129, 138, 139, 88, 136,
48, 139, 88, 131, 131, 75, 17, 118, 113, 118, 87, 95, 120, 95,
40, 120, 94, 93, 91, 70, 129, 122, 112, 102, 62, 143, 66, 57,
126, 120, 109, 95, 97, 126, 130, 97, 143, 117, 117, 74, 117,
117, 142, 98, 116, 94, 143, 145, 88, 108, 95, 108, 128, 150,
102, 124, 108, 132, 114, 134, 120, 149), c(0, 1, 1, 1, 1, 1,
2, 2, 3, 5, 6, 8, 9, 12, 13, 13, 14, 17, 20, 20, 22, 23, 25,
26, 28, 30, 31, 32, 32, 38, 39, 40, 42, 42, 43, 47, 47, 49, 55,
57, 57, 57, 57, 58, 62, 64, 66, 69, 69, 74, 75, 77, 77, 84, 84,
85, 86, 86, 87, 88, 91, 93, 94, 99, 102, 112, 113, 114, 118,
124), c(1, 0, 14, 22, 8, 35, 32, 34, 27, 47, 13, 18, 6, 10, 56,
26, 25, 24, 53, 19, 21, 39, 58, 40, 43, 51, 31, 62, 2, 57, 59,
64, 38, 30, 16, 66, 52, 45, 46, 48, 49, 15, 17, 20, 23, 37, 68,
29, 63, 36, 41, 60, 3, 28, 42, 11, 12, 65, 67, 7, 4, 5, 9, 50,
33, 44, 54, 55, 69, 61), c(0, 1, 2, 3, 4, 5, 6, 7, 8, 9, 10,
11, 12, 13, 14, 15, 16, 17, 18, 19, 20, 21, 22, 23, 24, 25, 26,
27, 28, 29, 30, 31, 32, 33, 34, 35, 36, 37, 38, 39, 40, 41, 42,
43, 44, 45, 46, 47, 48, 49, 50, 51, 52, 53, 54, 55, 56, 57, 58,
59, 60, 61, 62, 63, 64, 65, 66, 67, 68, 69), c(0, 0, 0, 0, 0,
0, 0, 0, 0, 0, 1, 1, 1, 1, 1, 1, 1, 2, 3, 3, 3, 3, 3, 3, 3, 3,
3, 3, 3, 3, 3, 3, 3, 3, 3, 3, 3, 3, 3, 3, 4, 4, 4, 4, 4, 4,
4, 4, 5, 5, 5, 5, 5, 5, 5, 5, 5, 6, 6, 6, 6, 6, 7, 7, 7, 7, 8,
8, 8, 8, 9, 9, 9, 9, 10, 11, 11, 11, 11, 11, 11, 11, 11, 11,
11, 11, 11, 12, 15, 15, 15, 16, 16, 17, 19, 23, 23, 25, 26, 26,
```

```
26, 26, 28, 28, 29, 29, 29, 29, 32, 33, 33, 33, 34, 35, 36, 36,
37, 41, 43, 43, 47, 47, 48, 48, 49, 49, 51, 51, 52, 54, 55, 57,
58, 58, 59, 59, 60, 60, 61, 63, 63, 63, 64, 67, 67, 68, 68, 68,
68, 69, 70, 70, 70, 70, 70, 70), c(0, 1, 6, 8, 9, 9, 10, 11,
11, 12, 13, 13, 13, 14, 16, 17, 17, 17, 18, 18, 18, 20, 20, 21,
22, 22, 23, 24, 24, 25, 25, 26, 27, 29, 29, 29, 29, 29, 29, 30,
31, 32, 32, 34, 35, 35, 35, 35, 37, 37, 38, 38, 38, 38, 38, 38,
39, 39, 43, 44, 44, 44, 44, 45, 45, 46, 46, 47, 47, 47, 49, 49,
49, 49, 49, 50, 51, 51, 53, 53, 53, 53, 53, 53, 53, 55, 56, 58,
59, 60, 60, 60, 61, 61, 62, 63, 63, 63, 63, 63, 64, 64, 64, 65,
65, 65, 65, 65, 65, 65, 65, 65, 65, 66, 67, 68, 68, 68, 68, 69,
69, 69, 69, 69, 69, 70, 70, 70, 70, 70, 70, 70, 70, 70, 70, 70,
70, 70, 70, 70, 70, 70, 70, 70, 70, 70, 70, 70, 70, 70, 70, 70,
70, 70, 70, 70, 70), list(c(1, 0, 1), structure(list(), .Names = character(0)),
  list(name = c("CA1018", "CA1047", "CA1067", "CA1080", "CA1093",
  "CA1101", "CA1108", "CA1137", "CA1147", "CA1177", "CA1604",
  "CA1619", "CA1627", "CA1644", "CA1647", "CA1649", "CA1662",
  "CA1663", "CA1664", "CA1674", "CA1676", "CA1683", "CA1687",
  "CA1694", "CA1696", "CA2628", "CA2629", "CA2651", "CA2653",
  "CA2665", "CA2666", "CA2686", "CA2691", "CA2703", "CA2704",
  "CA2706", "CA2709", "CA2714", "CA2715", "CA2718", "CA2721",
  "CA2729", "CA2738", "CA2745", "CA2753", "CA2757", "CA2762",
  "CA2765", "CA2769", "CA2776", "CA2780", "CA2782", "CA2783",
  "CA3003", "CA3010", "CA3015", "CA3022", "CA3026", "CA3027",
  "CA3036", "CA3049", "CA3057", "CA3058", "CA3060", "CA3064",
  "CA3065", "CA3066", "CA3071", "CA3076", "CA3082", "CA3084",
  "CA3085", "CA3121", "CA3130", "CA3139", "CA3141", "CA3143",
  "CA3145", "CA3146", "CA3154", "CA3174", "CA3405", "CA3417",
  "CA3419", "CA3424", "CA3425", "CA3427", "CA3432", "CA3460",
  "CA3470", "CA3480", "CA3607", "CA3612", "CA3616", "CA3617",
  "CA3621", "CA3622", "CA3638", "CA3640", "CA3655", "CA3659",
  "CA3662", "CA3665", "CA3666", "CA3668", "CA3680", "CA3695",
  "CA3708", "CA3753", "CA3754", "CA3765", "CA3769", "CA3774",
  "CA3787", "CA3789", "CA3792", "CA3793", "CA3801", "CA3807",
  "CA3826", "CA3838", "CA3850", "CA3854", "CA3868", "CA3869",
  "CA3879", "CA3901", "CA3911", "CA3934", "CA3949", "CA3956",
  "CA3964", "CA4006", "CA4010", "CA4022", "CA4036", "CA4039",
  "CA4050", "CA4058", "CA4059", "CA4077", "CA4088", "CA4095",
  "CA4100", "CA4102", "CA4166", "CA4175", "CA4200", "CA4215",
  "CA4333", "CA4382", "CA4383", "CA4390", "CA4587", "CB0009",
  "GX.MX|LX.WX"), `TRUE` = c("CA1018", "CA1047", "CA1067",
  "CA1080", "CA1093", "CA1101", "CA1108", "CA1137", "CA1147",
  "CA1177", "CA1604", "CA1619", "CA1627", "CA1644", "CA1647",
  "CA1649", "CA1662", "CA1663", "CA1664", "CA1674", "CA1676",
  "CA1683", "CA1687", "CA1694", "CA1696", "CA2628", "CA2629",
  "CA2651", "CA2653", "CA2665", "CA2666", "CA2686", "CA2691",
  "CA2703", "CA2704", "CA2706", "CA2709", "CA2714", "CA2715",
```

```
"CA2718", "CA2721", "CA2729", "CA2738", "CA2745", "CA2753",
"CA2757", "CA2762", "CA2765", "CA2769", "CA2776", "CA2780",
"CA2782", "CA2783", "CA3003", "CA3010", "CA3015", "CA3022",
"CA3026", "CA3027", "CA3036", "CA3049", "CA3057", "CA3058",
"CA3060", "CA3064", "CA3065", "CA3066", "CA3071", "CA3076",
"CA3082", "CA3084", "CA3085", "CA3121", "CA3130", "CA3139",
"CA3141", "CA3143", "CA3145", "CA3146", "CA3154", "CA3174",
"CA3405", "CA3417", "CA3419", "CA3424", "CA3425", "CA3427",
"CA3432", "CA3460", "CA3470", "CA3480", "CA3607", "CA3612",
"CA3616", "CA3617", "CA3621", "CA3622", "CA3638", "CA3640",
"CA3655", "CA3659", "CA3662", "CA3665", "CA3666", "CA3668",
"CA3680", "CA3695", "CA3708", "CA3753", "CA3754", "CA3765",
"CA3769", "CA3774", "CA3787", "CA3789", "CA3792", "CA3793",
"CA3801", "CA3807", "CA3826", "CA3838", "CA3850", "CA3854",
"CA3868", "CA3869", "CA3879", "CA3901", "CA3911", "CA3934",
"CA3949", "CA3956", "CA3964", "CA4006", "CA4010", "CA4022",
"CA4036", "CA4039", "CA4050", "CA4058", "CA4059", "CA4077",
"CA4088", "CA4095", "CA4100", "CA4102", "CA4166", "CA4175",
"CA4200", "CA4215", "CA4333", "CA4382", "CA4383", "CA4390",
"CA4587", "CB0009", "GX.MX|LX.WX"), sex = c(1L, 2L, 1L, 1L,
2L, 2L, 2L, 1L, 2L, 1L, 1L, 2L, 1L, 1L, 2L, 1L, 1L, 2L, 2L,
1L, 2L, 1L, 2L, 2L, 2L, 1L, 1L, 2L, 2L, 2L, 2L, 2L, 1L, 1L,
2L, 2L, 1L, 2L, 1L, 1L, 1L, 2L, 1L, 2L, 1L, 1L, 1L, 1L, 2L,
2L, 1L, 2L, 2L, 1L, 1L, 2L, 2L, 2L, 2L, 2L, 1L, 1L, 2L, 1L,
1L, 2L, 2L, 1L, 1L, 2L, 2L, 1L, 1L, 2L, 1L, 1L, 2L, 2L, 1L,
1L, 2L, 1L, 1L, 1L, 1L, 1L, 1L, 1L, 2L, 2L, 1L, 1L, 2L, 2L,
2L, 1L, 2L, 2L, 2L, 1L, 1L, 2L, 1L, 2L, 1L, 1L, 1L, 1L, 2L,
1L, 1L, 2L, 2L, 1L, 2L, 2L, 1L, 1L, 1L, 2L, 2L, 1L, 2L, 1L,
2L, 2L, 1L, 1L, 2L, 2L, 2L, 2L, 1L, 2L, 1L, 1L, 2L, 2L, 2L,
1L, 2L, 2L, 2L, 1L, 1L, 2L, 2L, 1L, 2L, 2L, 1L, 2L, 1L, 1L,
1L, 2L), cols = c("white", "white", "white", "white", "white",
"white", "white", "white", "white", "white", "white", "white",
"white", "white", "white", "white", "white", "white", "white",
"white", "white", "white", "white", "white", "white", "white",
"white", "white", "white", "white", "white", "white", "white",
"white", "white", "white", "white", "white", "white", "white",
"white", "white", "white", "white", "white", "white", "white",
"white", "white", "white", "white", "white", "white", "white",
"white", "white", "white", "white", "white", "white", "white",
"white", "white", "white", "white", "white", "white", "white",
"white", "white", "white", "white", "white", "white", "white",
"white", "white", "white", "white", "white", "white", "white",
"white", "white", "white", "white", "white", "white", "white",
"white", "white", "white", "white", "white", "white", "white",
"white", "white", "white", "white", "white", "white", "white",
"white", "white", "white", "white", "white", "white", "white",
"white", "white", "white", "white", "white", "white", "white",
"white", "white", "white", "white", "white", "white", "white",
```

```
    "white", "white", "white", "white", "white", "white", "white",
    "white", "white", "white", "white", "white", "white", "white",
    "white", "white", "white", "white", "white", "white", "white",
    "white", "white", "white", "white", "white", "white", "white",
    "white", "white", "white", "white", "white", "white", "white",
    "white", "white", "white", "white")), list(weight = c(0.333333333333333,
    0.0344827586206897, 0.0869565217391304, 0.0434782608695652,
    0.0333333333333333, 0.291666666666667, 0.166666666666667,
    0.0625, 0.2, 0.0909090909090909, 0.111111111111111, 0.0909090909090909,
    0.111111111111111, 0.0909090909090909, 0.142857142857143,
    0.0714285714285714, 0.0769230769230769, 0.0833333333333333,
    0.166666666666667, 0.0769230769230769, 0.0666666666666667,
    0.166666666666667, 0.25, 0.0625, 0.166666666666667, 0.166666666666667,
    0.0588235294117647, 0.04, 0.0769230769230769, 0.25, 0.125,
    0.111111111111111, 0.2, 0.111111111111111, 0.0714285714285714,
    0.166666666666667, 0.333333333333333, 0.0714285714285714,
    0.0666666666666667, 0.0833333333333333, 0.142857142857143,
    0.166666666666667, 0.2, 0.25, 0.0909090909090909, 0.1, 0.263157894736842,
    0.222222222222222, 0.0625, 0.166666666666667, 0.25, 0.0833333333333333,
    0.166666666666667, 0.1, 0.0833333333333333, 0.2, 0.111111111111111,
    0.166666666666667, 0.125, 0.0714285714285714, 0.05, 0.1,
    0.0909090909090909, 0.0909090909090909, 0.2, 0.0909090909090909,
    0.0909090909090909, 0.0454545454545455, 0.0952380952380952,
    0.111111111111111)))), class = "igraph")


# plot
par(mar=c(0.5,0.5,0.5,0.5))
par(mfrow = c(2,2))
plot(g2014, vertex.label = "",
        vertex.size =  degree(g2014)+2,
        vertex.color = V(g2014)$cols,
        edge.width = (E(g2014)$weight * 5))
plot(g2015, vertex.label = "",
        vertex.size =  degree(g2015)+2,
        vertex.color = V(g2015)$cols,
        edge.width = (E(g2015)$weight * 5))
plot(g2017, vertex.label = "",
        vertex.size =  degree(g2017)+2,
        vertex.color = V(g2017)$cols,
        edge.width = (E(g2017)$weight * 5))
plot(g2018, vertex.label = "",
        vertex.size =  degree(g2018)+2,
        vertex.color = V(g2018)$cols,
        edge.width = (E(g2018)$weight * 5))
```
